# Plastome-based phylogeny and biogeography of *Lactuca* L. (Asteraceae) support revised lettuce gene pool categories

**DOI:** 10.3389/fpls.2022.978417

**Published:** 2022-10-12

**Authors:** Ran Chu, Xuemin Xu, Zhenwei Lu, Yonggui Ma, Han Cheng, Shixin Zhu, Freek T. Bakker, M. Eric Schranz, Zhen Wei

**Affiliations:** ^1^ School of Life Sciences, Zhengzhou University, Zhengzhou, China; ^2^ School of Life Sciences, Qinghai Normal University, Xining, China; ^3^ Key Laboratory of Medicinal Animal and Plant Resources of Qinghai-Tibetan Plateau in Qinghai Province, Qinghai Normal University, Xining, China; ^4^ Biosystematics Group, Wageningen University, Wageningen, Netherlands

**Keywords:** *Lactuca* phylogenetics, lettuce genetic resources, Lactucinae, Cichorieae, Compositae

## Abstract

This study generated and analyzed complete plastome and internal transcribed spacer (ITS) data of 46 *Lactuca* species, 13 African endemic (AE) *Lactuca* species, and 15 species from eight related genera in Lactucinae. The new plastome and nuclear ITS sequences were then used to reconstruct the phylogenetic relationships of *Lactuca* species. The whole-plastome data were used to estimate divergence time and ancestral area reconstruction of the identified major *Lactuca* lineages. The results showed that *Lactuca* species are generally similar in plastome size, Guanine and Cytosine (GC) content, gene structure, and categories, although crop lettuce (*Lactuca sativa* L.) and its gene pool relatives were found to have one unique pseudogene (*ψ ndhF*), and *accD*, *atpF*, *cemA*, *clpP*, and *rpl22* showed signs of positive selection. Our phylogenomic analysis demonstrated that *Lactuca* is monophyletic after excluding *Lactuca alatipes* Collett and Hemsl and AE *Lactuca* species. AE *Lactuca* species are morphologically distinct from core *Lactuca* lineage and need to be excluded from *Lactua*. The core *Lactuca* species most likely originated from Asia-Temperate W ~6.82 Mya and then dispersed globally and formed nine clades. Finally, the lettuce gene pool concept was amended according to the phylogenetic and historical biogeographic analyses. This study revised the circumscription of *Lactuca*, revealed robust phylogenetic relationships within the genus, and provided insights into Lactucinae phylogeny. The lettuce gene pool species could be used as potential genetic resources for lettuce breeding.

## 1 Introduction

*Lactuca* L. contains cultivated lettuce (*Lactuca sativa* L.) and its wild relatives and is the second largest (~66 species) in Lactucinae (Asteraceae) (Kilian et al., 2009). Lactucinae taxa have been considered as complex and difficult to classify due to variable morphological characteristics and uncertain limitations among genera. In the last decade, the phylogenetic, molecular dating, and biogeographic analyses of Lactucinae at the subtribe level, based on non-coding nuclear marker [A44, internal transcribed spacer (ITS), external transcribed spacer (ETS)] and plastid DNA regions, revealed the general relationships and divergence among *Lactuca* and its allied genera (Wang et al., 2013; Kilian et al., 2017a; Kilian et al., 2017b; Wei et al., 2017; Güzel et al., 2021).

*Lactuca* wild species are distributed in Asia, Europe, Africa, and America (Lebeda et al., 2004a; Lebeda et al., 2004b; Lebeda et al., 2019). Wang et al. (2013) used extensive sampling of Chinese-centered Lactucinae taxa, based on ITS and five plastid DNA regions, and identified six core lineages in the subtribe, named *Cicerbita* Wall., *Cicerbita* II, *Lactuca*, *Melanoseris* Decne., *Notoseris* Shih, and *Paraprenanthes* Chang ex Shih. The “*Cicerbita* II lineage” was taxonomically referred to as *Kovalevskiella* Kamelin in the following study of Lactucinae, and *Prenanthes purpurea* L. and *Lactuca triquetra* Benth. and Hook.f. were both found to be monotypic genera (Kilian et al., 2017a; Kilian et al., 2017b). *L. triquetra*, endemic to Lebanon and Cyprus, was then transferred from *Lactuca* and revised as *Astartoseris triquetra* (Labill.) N. Kilian, Hand, Hadjik., Christodoulou and M. Bou Dagher-Kharrat (Kilian et al., 2017a). Wei et al. (2017) found that *Lactuca* species were polyphyletic, and African endemic (AE) *Lactuca* species + Asian *Melanoseris bracteata* (Hook.f. and Thomson ex C.B.Clarke) N.Kilian were sisters to a large polytomy including *Lactuca* and *Melanoseris* species. A diversification study of the Lactucinae using ITS and five non-coding plastid regions illustrated the Sub-Paratethyan origin of the subtribe and indicated close relationships between native AE *Lactuca* species and *Melanoseris* lineages (Kilian et al., 2017b). Jones et al. (2018) studied Northern Hemisphere disjunctions of *Lactuca* with a focus on the allopolyploid group endemic to America, using nuclear ribosomal DNA (ETS and ITS), a low-copy nuclear marker (A44) and five non-coding plastid markers. The phylogeny and systematics of the Lactucinae in SW Asia were analyzed and discussed based on ITS and five plastid regions, although the Lactucinae branch had many polytomies (Güzel et al., 2021). Phylogenetic relationships within *Lactuca* (~66 species) and the circumscription of *Lactuca* among its related genera were so far not well-resolved because of polytomies in the phylogenetic trees (Wang et al., 2013; Kilian et al., 2017b; Güzel et al., 2021).

High-throughput sequencing technology has helped resolve the origin and domestication history of cultivated lettuce and its most closely related wild species (Wei et al., 2021). However, the circumscription of *Lactuca* and the delimitation among *Lactuca* and other related lineages in Lactucinae were uncertain, and the phylogenetic relationships constructed using non-coding nrDNA or plastid regions were lacking robust support. A well-resolved phylogenetic tree could also help to identify lettuce gene pool species, meaning wild *Lactuca* could hybridize with cultivated lettuce (*L. sativa*) and generate fertile/sterile offspring or be partly cross-fertile with the crop (Zohary, 1991; van Treuren et al., 2012). In this study, we newly sampled plastomes of 46 wild *Lactuca* species plus 13 AE *Lactuca* species containing all of the biogeographic groups. Using these data, this paper aims to address four topics: 1) the general structure and characteristics of lettuce plastomes and the positive selection of plastid protein coding sequences (CDSs); 2) the taxonomic position and the circumscription of *Lactuca* and the main phylogenetic groups within the genus; 3) the origin of *Lactuca* and the historical biogeography of its ingroups; and 4) lettuce gene pool category revision.

## 2 Materials and methods

### 2.1 Plant materials and taxon sampling

**Figure 1:**
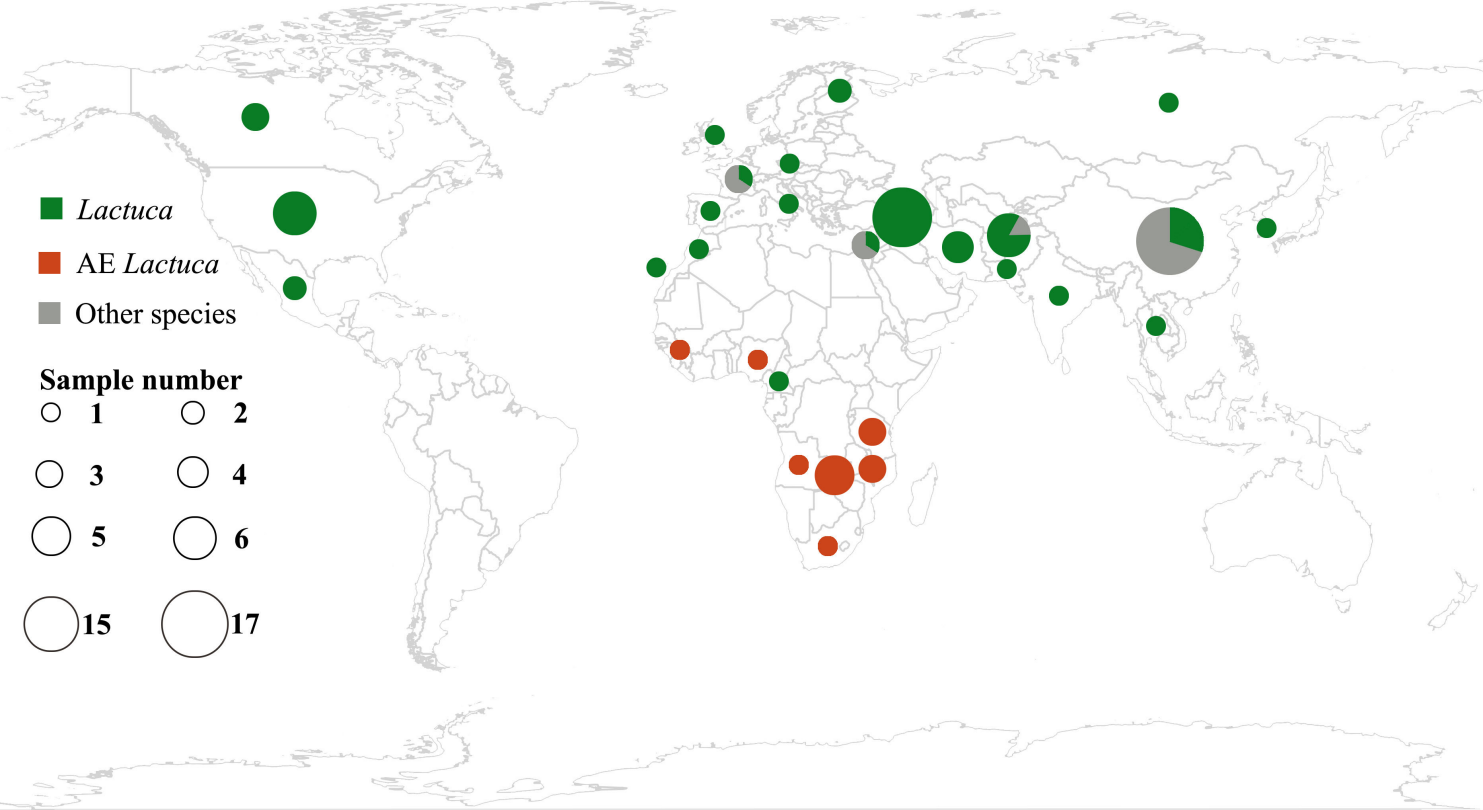
Geographic distribution of samples of Lactuca and related species using R package “rworldmap” (https://cran.r-project.org/web/packages/rworldmap/) and “ggplot2” (https://cran.r-project.org/web/packages/ggplot2/).

Plant leaf materials were mainly collected from international herbaria, including B, BM, CSH, K, E, PE, WAG and ZZU (**Figure 1**; **Supplementary Table S1**; herbarium code referring to Index Herbariorum, http://sweetgum.nybg.org/science/ih/). All of the necessary permissions for using herbarium accessions were approved by the respective curators. Two fresh-collected leaf samples of the Centre for Genetic Resources, Netherlands (**Supplementary Table S1**), were provided by Dr. MJW (Marieke) Jeuken (Wageningen University).

This study follows the concept of *Lactuca* and Lactucinae as indicated in the Cichorieae Portal (Kilian et al., 2009). After excluding species with ambiguous concepts or description, we identified 66 *Lactuca* species. Our *Lactuca* sampling (46 species) thus covers 70% of the genus and all geographical groups (Lebeda et al., 2004b; Kilian et al., 2017b). We also included species from eight related Lactucinae lineages: *Astartoseris*, *Cicerbita*, *Faberia* Hemsl. ex F. B. Forbes and Hemsl, *Kovalevskiella* [*Cicerbita auriculiformis* (C. Shih) N. Kilian and *Cicerbita azurea* (Ledeb.) Beauverd], *Melanoseris* [*Melanoseris decipiens* (C.B.Clarke) N.Kilian and Ze H.Wang and *Lactuca crambifolia* Boiss.], *Notoseris*, *Paraprenanthes*, and *Prenanthes* (Kilian et al., 2017b). In the taxon sampling, four complete plastomes were downloaded from NCBI, nine from China National GeneBank DataBase (https://db.cngb.org/), and two partial cp genomes were from a previous publication (Pouchon et al., 2018) (**Supplementary Table S2**). In total, this study comprised 111 accessions (96 of which are new), representing a total of 74 Lactucinae species and three outgroups (one species in Cichoriinae and two in Scorzonerinae) (**Supplementary Tables S1**, **S2**).

### 2.2 DNA isolation and purification

Total DNA was extracted from herbarium leaf material using a modified cetyltrimethyl-ammonium-bromide (CTAB) method (Doyle and Doyle, 1987) and purified with Wizard DNA Clean-Up System (Promega Corp.) (Wei et al., 2017). In total, DNA extracts were successfully obtained from 96 *Lactuca* and related taxa (**Supplementary Table S1**).

### 2.3 Sequencing, data filtering, assembling, and annotation of plastomes

Eight-four DNA extracts were sequenced using Illumina HiSeq Xten/4000/Novaseq platforms (Novogene Inc., Tianjin, China). The other 12 samples were sequenced by the National High-Throughput DNA Sequencing Centre of the University of Copenhagen using Illumina HiSeq 2000 platform (http://seqcenter.ku.dk/facilities/). The clean data were assembled using NOVOPlasty v4.0 (Dierckxsens et al., 2016) with references AP007232 and DQ383816 and Get Organelle e-1.7.3.2 (Jin et al., 2020) with default settings and angiosperm plastome references. The assembled cp genomes were annotated and manually corrected in Geneious Prime 2020.1.2 (https://www.geneious.com). The annotated tRNA genes were checked using the tRNAscan-SE 2.0 Web Server (Chan et al., 2021). The physical maps of the cp genomes were visualized using OrganellarGenomeDRAW (Greiner et al., 2019). The same clean data of 96 samples were also used to assemble ITS sequences using Get Organelle e-1.7.3.2 (Jin et al., 2020) with reference ITS sequences of *L. serriola* (LT721932), *L. georgica* (LT722049), and *L. altaica* (LT722033). The ITS DNA regions were then extracted from the assembled sequences. Thirty-two previously published ITS sequences (Wang et al., 2013; Kilian et al., 2017b; Güzel et al., 2021) were downloaded and added to the phylogenetic analysis (**Supplementary Table S3**).

### 2.4 Plastome comparisons and analyses

The CDSs of 108 plastomes of Lactucinae species were extracted and aligned using Geneious Prime 2022.1.1 (https://www.geneious.com). The variation rates, conserved sites, parsimony informative sites, singleton sites, and variation frequency were calculated using MEGA X (Kumar et al., 2018). The plastomes of *Lactuca* and AE *Lactuca* (59 species, one sequence per species) were uploaded to mVISTA, and the alignment and variation were visualized with Shuffle-LAGAN mode (Mayor et al., 2000). The annotation of *L. sativa* (AP007232) was used as a reference. The cp genes of *Lactuca* and AE *Lactuca* (59 species, one sequence per species) near the boundaries of the large single-copy region (LSC), small single-copy region (SSC), and two inverted repeat regions (IRs) were visualized and compared using IRscope (https://irscope.shinyapps.io/irapp/) (Amiryousefi et al., 2018).

### 2.5 Phylogenetic analyses

The SSC inversions in plastomes were reversed first and then aligned with all plastome sequences in MAFFT version 7 (https://mafft.cbrc.jp/alignment/software/). *Gelasia hirsuta* (Gouan) Zaika, Sukhor. and N.Kilian, *Pseudopodospermum hispanicum* (L.) Zaika, Sukhor. and N.Kilian of Scorzonerinae and *Cichorium intybus* L. of Cichoriinae were used as outgroups. The phylogenetic analyses, using whole-plastome sequences, were constructed by maximum likelihood (ML) and Bayesian inference (BI) methods in CIPRES Science Gateway using the tool of RAxML-HPC2 on XSEDE 8.2.12 and MrBayes on XSEDE 3.2.7a (https://www.phylo.org/portal2/login!input.action). The RAxML tree was inferred with 1,000 bootstrap replicates using the GTR + GAMMA model for the nucleotide datasets; “print branch length (-k)” under “Configure bootstrapping” and other parameters used default settings. The parameters of BI analyses in MrBayes were as follows: lset nst = 6 rates = gamma; unlink statefreq = (all) revmat = (all) shape = (all) pinvar = (all); prset applyto = (all) ratepr = variable; mcmcp ngen = 10000000, relburnin = yes, burninfrac = 0.25, printfreq = 1000, samplefreq = 10000, nchains = 4 temp = 0.05. The burn-ins and effective sampling sizes (ESSs) of all parameters were checked for convergence between simultaneous runs in Tracer v.1.7.1 (Rambaut et al., 2018).

The rDNA ITS sequences from *Lactuca* and related species were also used for constructing phylogenetic trees following the same described methods as above for plastomes. All new ITS sequences were submitted to GenBank (https://www.ncbi.nlm.nih.gov/genbank/) under accession number OP070064–OP070156. FigTree 1.4.4 (http://tree.bio.ed.ac.uk/software/figtree/) and iTOL v6 (Letunic and Bork, 2021) were used to visualize and edit the output trees.

The chromosome number data were downloaded and collected from the Chromosome Counts Database (http://ccdb.tau.ac.il/) and previous publications (Jones et al., 2018). The pseudogenes were manually checked and recorded in Geneious Prime 2022.1.1 (https://www.geneious.com). Chromosome numbers and the pseudogenes of *Lactuca* and related species were identified, analyzed, and plotted on the phylogenetic tree.

### 2.6 Positive selection analysis

Selection pressures on the plastid CDS of 72 *Lactuca* taxa (representing 45 species) were analyzed in Datamonkey 2.0 (http://www.datamonkey.org/) using Single-Likelihood Ancestor Counting method with universal code (Sergei et al., 2005; Weaver et al., 2018). *Lactuca alatipes* Collett and Hemsl. was excluded in this analysis due to its phylogenetic position outside *Lactuca* in our phylogenetic analyses (see *Results*). The CDSs of 12 lettuce gene pool species (representing 23 taxa) (Zohary, 1991; van Treuren et al., 2012), *L. sativa*, *Lactuca altaica* Fisch. and C.A.Mey., *Lactuca aculeata* Boiss., *Lactuca serriola* L., *Lactuca georgica* Grossh., *Lactuca scarioloides* Boiss., *Lactuca virosa* L., *Lactuca saligna* L., *Lactuca viminea* (L.) J.Presl and C.Presl, *Lactuca reviersii* Litard. and Maire, *Lactuca tetrantha* B.L.Burtt and P.H.Davis, and *Lactuca orientalis* Boiss., were also checked for selection pressure analysis. After excluding pseudogenes, 61 plastid CDSs shared by all of the *Lactuca* taxa and 73 plastid CDSs shared by lettuce gene pool taxa were used in the analyses.

### 2.7 Divergence time estimation

A total of 78 complete cp genomes (each plastome representing one species) of *Lactuca* and its related lineages were used in the divergence time estimation. The age of *Lactuca* and Lactucineae taxa was calculated with the Strict Clock model and GTR + Gamma site model in BEAST v2.6.0 (Bouckaert et al., 2019). The Calibrated Yule Model was selected as tree priors. Three nodes were calibrated: 1) The oldest reported Cichorieae fossil (early Miocene, 22.0–28.4 Mya) of the *C. intybus* type pollen, distributed widely in all Cichorieae subtribes except Scorzonerinae (Hochuli, 1978; Tremetsberger et al., 2012; Kilian et al., 2017b); 2) The secondary calibration prior was selected for the stem node of *Lactuca* lineage [8.0 Mya, 95% highest posterior density (95% HPD) 5.8–10.2 Mya] (Kilian et al., 2017b); 3) The third calibration prior was set for a group of four species (*Lactuca intricata* Boiss., *Lactuca undulata* Ledeb., *Lactuca glauciifolia* Boiss., and *Lactuca perennis* L.) within *Lactuca* (4.4 Mya) from Kilian et al. (2017b) calculated by plastid genes.

The Monte Carlo Markov chains (MCMCs) were run for 100,000,000 generations, and the tree was set to sample every 10,000 generations. The first 10% of trees were discarded as burn-ins. The burn-ins and ESSs of all parameters were checked for convergence between simultaneous runs in Tracer v.1.7.1 (Rambaut et al., 2018). The trees were resampled at lower frequency of 30,000 using LogCombiner in BEAST v2.6.0 (Bouckaert et al., 2019). TreeAnnotator in BEAST was used to summarize the most probable trees into a maximum clade credibility (MCC) tree with median node heights and visualized in FigTree v1.4.4 (http://tree.bio.ed.ac.uk/software/figtree/).

### 2.8 Historical biogeographic reconstruction

The geographic information of taxon distribution was downloaded from the Global Biodiversity Information Facility (GBIF, https://www.gbif.org/) database using R package “dismo” (https://cran.r-project.org/web/packages/dismo/) and compared with the native distribution of *Lactuca* on the Cichorieae Portal (Kilian et al., 2009). The ancestral area reconstruction was estimated using native distribution data in RASP V4.2 (Yu et al., 2020), and the Statistical Dispersal-Vicariance-Analysis (S-DIVA) model was used to reconstruct the ancestral range of each node using the tree files generated by BEAST v2.6.0 (Bouckaert et al., 2019). The regions of *Lactuca* and its related genera were counted and modified from Kilian et al. (2017b) and Jones et al. (2018) (A: N + Central + E Europe, B: SW +SE Europe, C: N Africa + Macaronesia, D: Tropical Africa, E: S Africa, F: Asia-Temperate W, G: Siberia + Russian Far East, H: Asia-Temperate E, I: Indian Subcontinent, J: Asia-Tropical E, K: N America, L: S America).

## 3 Results

### 3.1 Comparative analysis of plastomes of *Lactuca* and related species

**Figure 2:**
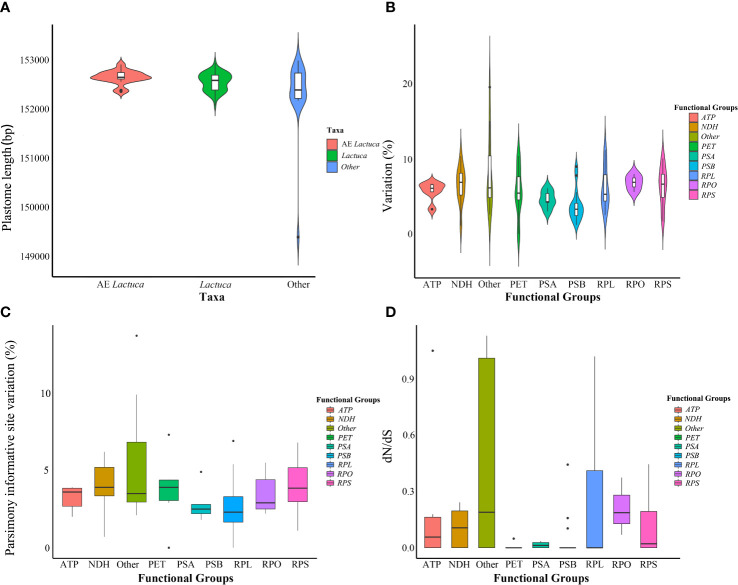
Information on plastomes of *Lactuca* and African endemic *Lactuca* species. **(A)** Plastome length. **(B)** Variation of different functional groups. **(C)** Parsimony informative site variation of different functional groups. **(D)** Non-synonymous/synonymous rate ratios (dN/dS) of protein-coding genes of different functional groups in cultivated lettuce gene pool species.

This study newly sequenced and assembled 96 plastomes of *Lactuca* and related taxa (representing 74 species, see **Supplementary Table S1**). The length of complete plastomes of *Lactuca* species (including native African ones) ranges from 152,076 to 152,947 bp (**Figure 2A**; **Supplementary Figure S1**), and the sizes of LSC, SSC, and IR of *Lactuca* species (including native African ones) are from 83,469 to 84,271 bp, 18,326 to 18,780 bp, and 24,849 to 25,106 bp (**Supplementary Table S4**). The GC contents of *Lactuca* species (including native African ones) are stable, ranging from 37.5% to 37.7% (**Supplementary Table S4**). The number of plastid genes of *Lactuca* species (including native African species) is from 126 to 134 due to the presence of pseudogenes. The information on pseudogenes was recorded and classified (**Supplementary Table S5**) for further comparison with phylogenetic analyses. The number of rRNA (8) and tRNA (37) genes were consistent across all taxa (**Supplementary Table S4**). Other Lactucinae species have similar cp genome lengths, GC contents, and numbers of genes (**Supplementary Table S4**).

Minor variations in plastome length of *Lactuca* and AE *Lactuca* (one sequence per species, in total 59 species) were observed, mostly in the SSC. Whereas it was relatively conserved across the IRs (**Supplementary Figure S2**). Photosynthesis-related genes were the most conserved genes, whereas *ycf1*, *matK*, *accD*, *rpl22*, *ccsA*, and *rpl36* had the highest variation (**Figure 2B**). The genes with the largest number of parsimoniously informative sites were *ycf1*, *rpoC2*, *matK*, *ycf2*, *ndhF*, *accD*, and *rpoB* (**Figure 2C**).

The same *Lactuca* and AE *Lactuca* taxa were used for the boundary comparison of LSC, SSC, and two IRs. The results showed slight contraction and expansion of the genes near the junction sites. Thirty species (34 taxa), 13 of which were AE *Lactuca* species, had one inverted SSC region (**Supplementary Figure S3**). The gene number and type of plastomes of those species with inverted SSC were similar to other plastomes with regular SSC.

### 3.2 Phylogenetic relationships of *Lactuca* and relatives

**Figure 3:**
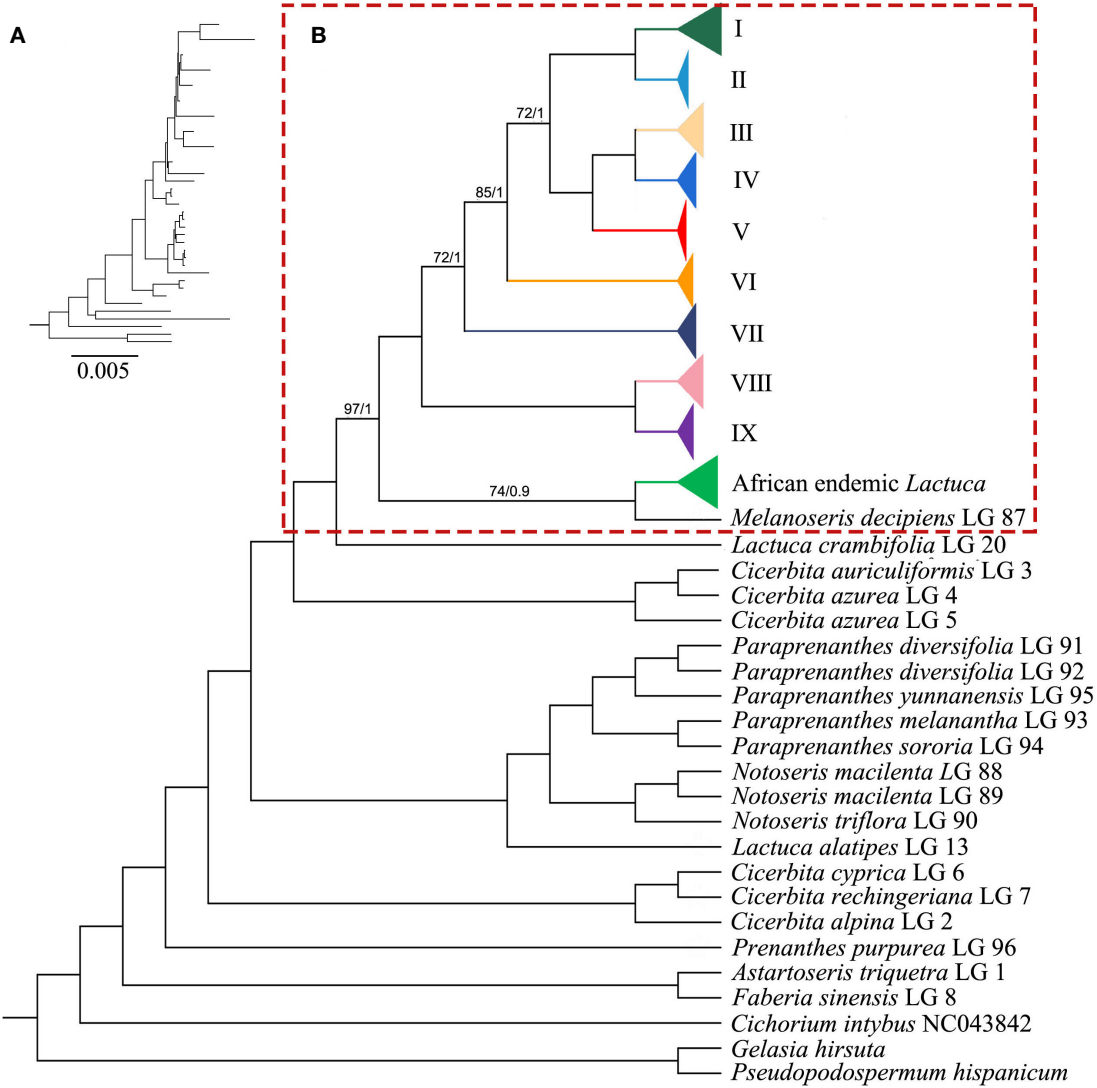
Phylogenetic cladogram of *Lactuca* and related genera based on complete plastomes. **(A)** Phylogenetic tree with branch length. **(B)** Phylogenetic cladogram with collapsed clades of *Lactuca* and AE *Lactuca* species. The width of the triangle represents the number of species. Support values are maximum likelihood bootstrap (BS) / Bayesian posterior probability (PP). No support values are shown for nodes with full support (BS = 100, PP = 1).

**Figure 4:**
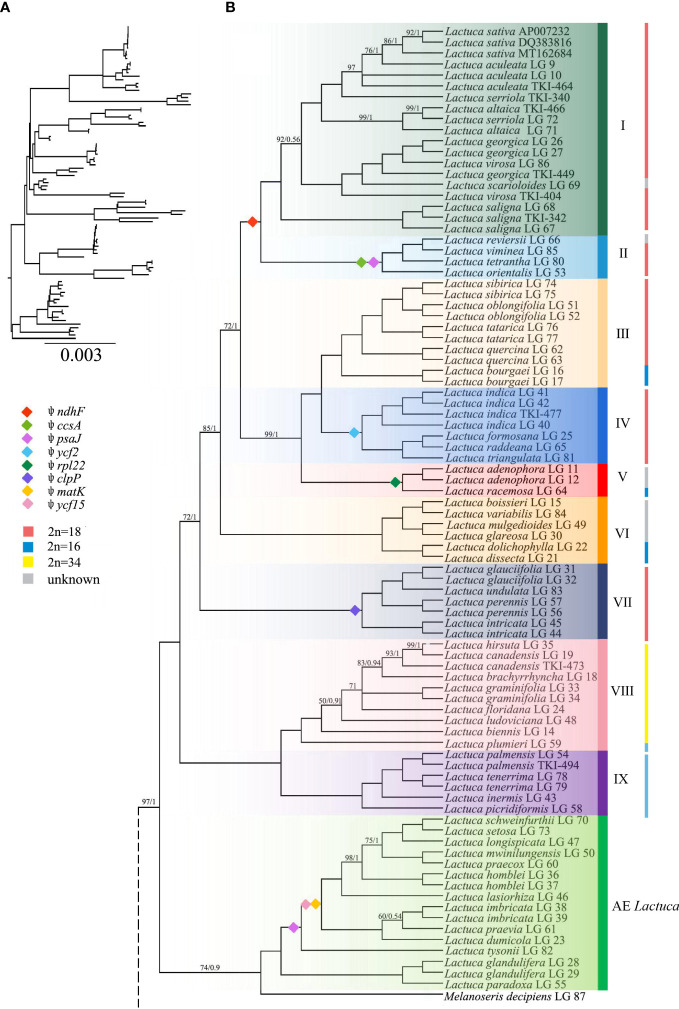
Phylogenetic tree of *Lactuca* and AE *Lactuca* species inferred from plastomes. **(A)** Phylogenetic tree with branch length. **(B)** ML/BI tree, the color is consistent with that in **Figure 3**. The nodes with full support (BS = 100, PP = 1) are not shown. The information on pseudogene and reverse SSC is indicated on the branches. The vertical line on the right represents the number of chromosomes. Support values are maximum likelihood bootstrap (BS) / Bayesian posterior probability (PP). No support values are shown for nodes with full support (BS = 100, PP = 1).

The ML and BI trees generally shared the same topologies (**Figures 3**, **4**). In the results, most of the nodes of the phylogenetic tree were fully supported with BS = 100 and PP = 1. Branches of the phylogenetic trees with bootstrap <50 were collapsed. Chromosome numbers and presence of pseudogenes of *Lactuca* and related species are indicated at the responding nodes (**Figures 3**, **4**).

The tree was rooted with three outgroup species [*G. hirsuta* (Scorzonerinae), *P. hispanicum* (Scorzonerinae), and *C. intybus* (Cichoriinae)]. In Lactucinae, *A. triquetra* and *Faberia sinensis* Hemsl. are closely related and sisters to other Lactucinae species. The monotypic *P. purpurea* is a sister to three *Cicerbita* species and five other Lactucinae lineages. *L. alatipes* is found to be related to *Notoseris* and *Paraprenanthes* species other than *Lactuca* species. We downloaded the plastid DNA sequences of *Paraprenanthes* species from a previous publication (Wang et al., 2013), in which *Lactuca parishii* Craib ex Hosseus LAC 028 was treated as *Paraprenanthes umbrosa* (Dunn) Sennikov but later revised as *P. parishii* (Kilian et al., 2017b) and reconstructed a RAxML tree (**Supplementary Figure S4**; **Supplementary Table S6**). Three *Kovalevskiella* taxa (*C. azurea* LG 4, *C. azurea* LG 5, and *C. auriculiformis* LG 3) are the sisters to taxa of *Melanoseris* (*L. crambifolia* and *M. decipiens*), the AE *Lactuca*, and the core *Lactuca* (Clades I–IX).

The *Melanoseris* lineage (sensu in Kilian et al., 2017b) member *L. crambifolia* is a sister to *M*. *decipiens* + AE *Lactuca* Clade (**Figure 4**), containing 16 samples (13 species) endemic to Africa, and all of them are sisters to the core *Lactuca* clade. Thirteen taxa in this clade have reversed SSC regions, excluding *Lactuca setosa* Stebbins ex Jeffrey, *Lactuca lasiorhiza* (Hoffm.) Jeffrey, and *Lactuca paradoxa* Sch.Bip. ex A.Rich. Thirteen AE *Lactuca* species (excluding *Lactuca glandulifera* Hook.f. and *L. paradoxa*) share a common pseudogene (*ψ psaJ*), and 12 taxa, excluding *L. glandulifera*, *L. paradoxa*, and *Lactuca tysonii* (E.Phillips) C.Jeffrey, share two pseudogenes (*ψ matK* and *ψ ycf15*).

The core *Lactuca* was identified to comprise nine clades (**Figures 3**, **4**). Clade IX and Clade VIII are sisters. Clade IX includes four species (six taxa) with a chromosome number of 16 (2n = 16). Clade VIII contains eight species, and seven of them are American native (especially N American endemic, NAE) *Lactuca* with a chromosome number of 34 (2n = 34). The only exception is *Lactuca plumieri* Gren. and Godr., distributed in Europe (2n = 16).

Most species in Clades I–VII have a chromosome number of 18 (2n = 18) despite some taxa with 16 or unknown chromosomes. The four *Lactuca* species (2n = 18) in Clade VII are sisters to all the other species in Clades I–VI. *L. perennis* LG 57, *L. intricata* LG 44, and *L. intricata* LG 45 have reverse SSC. Contraction and expansion of the junction sites of the cp genomes were observed in *L. perennis* LG 56, *L. perennis* LG 57, and *L. intricata* LG 44. Clades III, IV, V, and VI form a sister clade to a clade comprising Clade I (the crop lettuce clade) and Clade II. Clade VI contains six *Lactuca* species, and three of them have reverse SSC. *Lactuca dolichophylla* Kitam. LG 22 and *Lactuca dissecta* D.Don LG 21 are sister groups with the same chromosome number (2n = 16) and showed contraction and expansion near the junction sites of the cp genomes. *Lactuca variabilis* Bornm. LG 84, *Lactuca mulgedioides* Boiss. and Kotschy ex Boiss. LG 49, and *L. dissecta* LG 21 were observed to have reverse SSC.

The species in Clade V share one common pseudogene (*ψ rpl22*). *Lactuca racemosa* Willd. LG 64 (2n = 16) has a reversed SSC, and the genes near the junction sites of the cp genomes of *Lactuca adenophora* Boiss. and Kotschy LG 11 showed contraction to some extent. Clade IV includes four species (2n = 18), and they are sister groups to species in Clade III with mixed chromosome numbers (2n = 16, 18). In Clade IV, all of the species were *Pterocypsela* C.Shih and share a pseudogene (*ψ ycf2*), and *Lactuca indica* L. LG 41 has reverse SSC. In Clade III, *Lactuca bourgaei* Irish and Taylor LG 16 and LG 17 (2n = 16) are sisters to four other species (2n = 18). Some genes near the junction sites of the cp genomes of *Lactuca oblongifolia* Nutt. LG 51 and LG 52 and *L. tatarica* LG 76 and LG 77 showed expansion, and *L. oblongifolia* LG 51 has reverse SSC.

Clade I and Clade II are closely related with the same chromosome number (2n = 18). Clade II includes four species, and three have reverse SSC. All of the species share two pseudogenes, *ψ ccsA* and *ψ psaJ*. Clade I comprises 19 *Lactuca* taxa representing cultivated lettuce and its relatives. Three *L. saligna* accessions are sisters to all of the other *Lactuca* taxa in Clade I. *L. georgica*, *L. virosa*, and *L. scarioloides* are sisters to *L. serriola*, *L. altaica*, *L. sativa*, and *L. aculeata*. Reverse SSC was observed in *L. aculeata* LG 10, *L. sativa* DQ383816, *L. georgica* LG 26, and *L. saligna* LG 68.

### 3.3 The incongruence between plastid and internal transcribed spacer (ITS) phylogenetic trees

Ninety-three ITS sequences from 73 species were successfully assembled. In the end, a total of 125 ITS sequences from 105 *Lactuca* and related species were used for phylogenetic analysis. The taxa sampling included 53 *Lactuca* species, accounting for 80.3% of the genus with a higher coverage than cp trees. However, many nodes of the ML tree support values were lower than 50%, and the BI tree had many polytomies. Therefore, those branches with low node support values were collapsed for confidence (**Supplementary Figures S5**, **S6**). As results, the phylogenetic positions of Lactucinae genera were not well resolved. The systematic position of *P. purpurea* was outside Lactucinae, and *A. triquetra* plus *F. sinensis* were parallel to the core groups of Lactucinae. *Cicerbita*, *Kovalevskiella*, *Notoseris-Paraprenanthes*, *Melanoseris* + *L. bourgaei* + AE *Lactuca*, and core *Lactuca* were in a large polytomy. *L. alatipes* is a sister to *L. parishii* in ITS trees, while in plastid trees, it is a sister to *Notoseris-Paraprenanthes* clade. *L. bourgaei* is close to *Melanoseris* clade, consisting of *L. crambifolia* and *M. decipiens*, but in chloroplast genome phylogenetic trees, it is in the core *Lactuca*.

Phylogenetic relationships of the core *Lactuca* lineages inferred from ITS sequences had similar clades as the plastome trees. The main incongruences between the two types of phylogenetic trees were in Clades III and VI. The two clades in the plastome tree were divided into two and three clades, respectively, in ITS trees. Clade III-1 is *Lactuca quercina* L., sister group to the crop lettuce and its relatives (Clade I). Clade III-2 contains *L. oblongifolia*, *Lactuca sibirica* Benth. ex Maxim., and *L. tatarica* and is a sister to Clade II. Clade III-3 is *L. bourgaei* within the *Melanoseris* Clade, a sister to AE *Lactuca* species. Clade VI was divided into two parts in ITS results, Clade VI-1 containing seven species and Clade VI-2 including *L. dolichophylla* and *L. dissecta*.

### 3.4 Positive selection of plastid CDSs

After excluding pseudogenes, 61 plastid CDSs shared by all of the *Lactuca* taxa and 73 plastid CDSs shared by lettuce gene pool taxa (Clade I and II) were used respectively to analyze the possible presence of positive selection. The results showed purifying selection on 61 common protein-coding genes of all *Lactuca* lineages (**Supplementary Figure S7**). The selection pressure analysis on 73 shared CDSs of the 12 lettuce gene pool species and cultivated lettuce demonstrated that five genes were under positive selection (dN/dS > 1), namely, *accD*, *atpF*, *cemA*, *clpP*, and *rpl22* (**Figure 2D** **;** **Supplementary Figure S8**).

### 3.5 Historical biogeographic estimation

**Figure 5:**
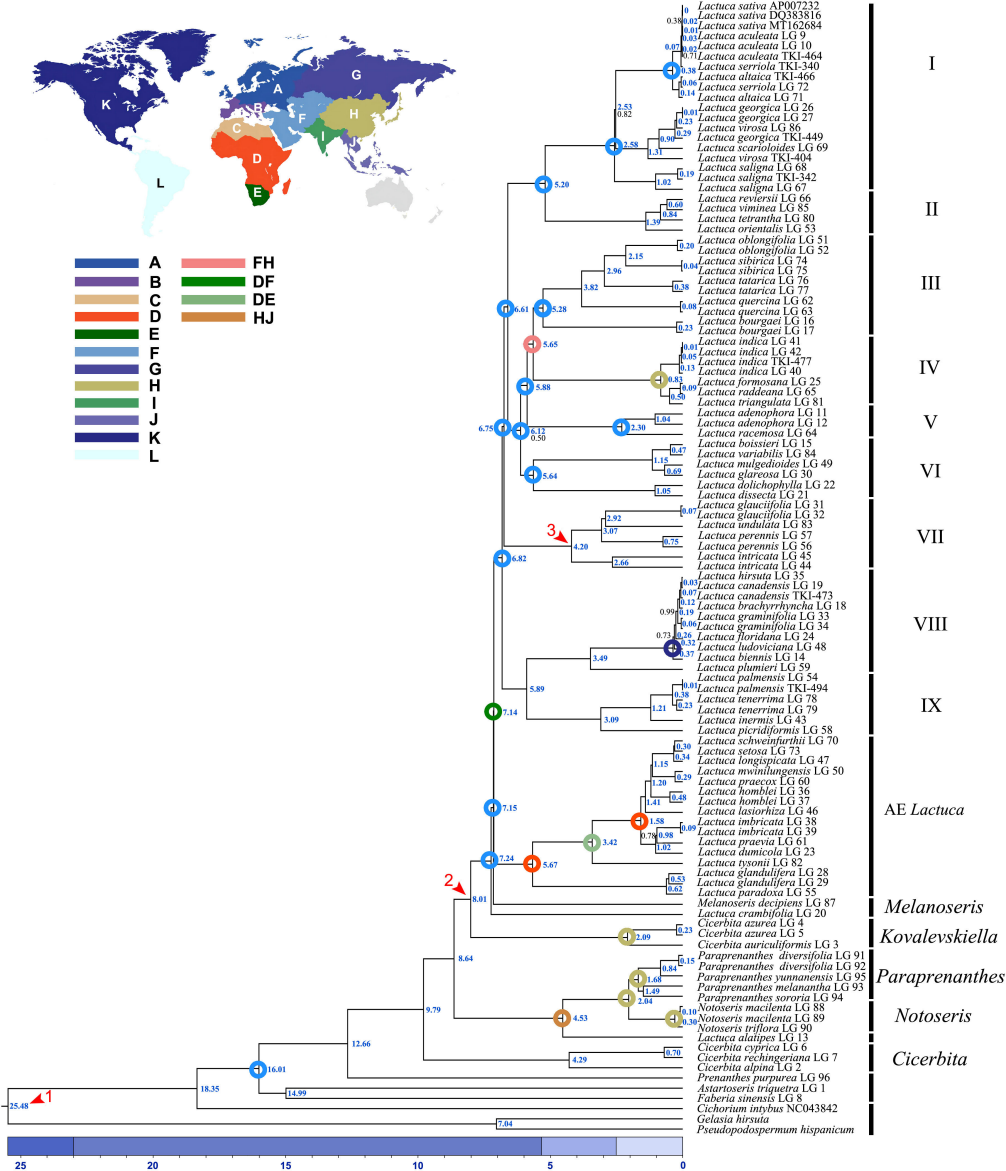
Dated phylogram of *Lactuca* and related genera based on complete plastomes. The red arrows are the calibration points of divergence time. The blue numbers indicate the median node age of divergence, and the black numbers indicate the support of Bayesian posterior probability (PP = 1 is not displayed; colored circles displayed on nodes are ancestral ranges with probability = 1, except the one of *Lactuca* lineages). Geographical areas are indicated with different colors: **(A)** N + Central + E Europe, **(B)** SW + SE Europe, **(C)** N Africa + Macaronesia, **(D)** Tropical Africa, **(E)** S Africa, **(F)** Asia-Temperate W, **(G)** Siberia + Russian Far East, **(H)** Asia-Temperate E, **(I)** Indian Subcontinent, **(J)** Asia-Tropical E, **(K)** N America, **(L)** S America.

As the phylogenetic trees generated using ITS sequences contained polytomies, we only did further divergence time analysis using cp genomes. The topologies of phylogenetic trees constructed based on cp genomes using BEAST, RAxML, and MrBayes were similar despite the incongruent positions of *M. decipiens* and Clade VI. In ML and BI trees, *M. decipiens* is the sister group to the AE *Lactuca* species (**Figure 3**), while it is the sister to all of the *Lactuca* lineages (including AE *Lactuca* taxa) in the BEAST-dated tree (**Figure 5**). Most of the nodes in the dated phylogram of *Lactuca* and its related genera in Lactucinae were fully supported (PP = 1). The most recent common ancestor (MRCA) of Lactucinae and Cichoriinae started diverging around 18.35 Mya (**Table 1**). The MRCA of Lactucinae existed around 16.01 Mya in Asia-Temperate W (PP = 1; **Figure 5**). The core Lactucinae (excluding *Astartoseris*, *Faberia*, and *Prenanthes*) was estimated to diverge around 9.79 Mya with an ancestral area of Asia-Temperate W (PP = 1). *Paraprenanthes* and *Notoseris* lineages share an MRCA from Asia-Temperate E (PP = 1) around 2.04 Mya (**Table 1**).

**Table 1:** Estimated divergence time of *Lactuca* and related genera based on the complete plastome sequences.

Clade	Node	Complete chloroplast genome tree		
		Posterior probability	Median node age (Mya)	95% Highest posterior density
Lactucinae	Stem	1	18.35	16.88 ~ 19.79
	Crown	1	16.01	14.72 ~ 17.27
Core Lactucinae	Stem	1	12.66	11.64 ~ 13.71
	Crown	1	9.79	8.97 ~ 10.55
*Cicerbita* lineage	Stem	1	9.79	8.97 ~ 10.55
	Crown	1	4.29	3.82 ~ 4.80
*Notoseris*-*Paraprenanthes* lineage	Stem	1	4.53	4.10 ~ 5.03
	Crown	1	2.04	1.77 ~ 2.28
*Kovalevskiella* lineage	Stem	1	8.01	7.32 ~ 8.63
	Crown	1	2.09	1.77 ~ 2.42
AE *Lactuca-Lactuca*	Stem	1	7.15	6.56 ~ 7.74
	Crown	1	7.14	6.55 ~ 7.73
*Lactuca* lineage	Stem	1	7.14	6.55 ~ 7.73
	Crown	1	6.82	6.27 ~ 7.40
Clade IX	Stem	1	5.89	5.40 ~ 6.39
	Crown	1	3.09	2.79 ~ 3.42
NAE *Lactuca*-*L. plumieri* (Clade VIII)	Stem	1	5.89	5.40 ~ 6.39
	Crown	1	3.49	3.09 ~ 3.85
Clade VII	Stem	1	6.75	6.19 ~ 7.31
	Crown	1	4.20	3.82 ~ 4.55
Clade VI	Stem	1	6.12	5.62 ~ 6.68
	Crown	1	5.64	5.04 ~ 6.22
Clade V	Stem	1	5.88	5.31 ~ 6.43
	Crown	1	2.30	2.00 ~ 2.60
Clade IV	Stem	1	5.65	5.08 ~ 6.15
	Crown	1	0.83	0.69 ~ 0.97
Clade III	Stem	1	5.65	5.08 ~ 6.15
	Crown	1	5.28	4.80 ~ 5.79
Clade I-II	Stem	1	6.61	6.04 ~ 7.16
	Crown	1	5.20	4.73 ~ 5.63
Clade II	Stem	1	5.20	4.73 ~ 5.63
	Crown	1	1.39	1.23 ~ 1.58
Clade I	Stem	1	5.20	4.73 ~ 5.63
	Crown	1	2.58	2.31 ~ 2.85

The divergence of the AE *Lactuca* and the core *Lactuca* lineage began from an MRCA distributed between Tropical Africa and Asia-Temperate W around 7.14 Mya. The MRCA of the AE *Lactuca* (**Figure 5**) very likely originated from Tropical Africa (PP = 1), and the recent speciation events occurred around 5.67 Mya (**Table 1**). After dispersal and vicariance events, the AE *Lactuca* lineage is now distributed in Tropical Africa (D) and S Africa (E).

Around 6.82 Mya, the non-African *Lactuca* lineage was inferred to have a possible MRCA from Asia-Temperate W (F). The *Lactuca* lineage then diffused to Europe, Asia, Indian Subcontinent, and North America after a series of dispersal, vicariance, and extinction events (**Supplementary Figure S9**; ancestral area estimation in **Supplementary Material 1**). *Lactuca* species in Clades I–VII share an MRCA originating from Asia-Temperate W with a probability of 1. The MRCA of the NAE *Lactuca* (excluding *L. oblongifolia*) and *L. plumieri* (Clade VIII) began to diverge around 3.49 Mya, and thereafter, the MRCA of NAE *Lactuca* diverged around 0.37 Mya and dispersed from N America to S America.

## 4 Discussion

In this study, we used 46 *Lactuca* species plus 13 AE *Lactuca* species from all geographic (European, Asian, African, and American) groups to reconstruct deep-level phylogenetic relationships and estimate the historical biogeography of *Lactuca*. We will discuss four main issues: 1) the plastid genome evolution of cultivated lettuce and wild *Lactuca* species; 2) the delimitation among *Lactuca* and other lineages in Lactucinae and AE *Lactuca* species; 3) the origin, phylogeny, and historical biogeography of *Lactuca* and the main phylogenetic ingroups; and 4) revision to the lettuce gene pool category.

### 4.1 The evolution of *Lactuca* plastomes

This study successfully sequenced, assembled, and annotated 96 complete plastomes of *Lactuca* lineages and related species. Generally speaking, the newly generated *Lactuca* plastomes showed similarities (Clade I to Clade IX) in plastome size, GC content, structure, and gene category, as well as published plastomes of other Asteraceae lineages (Pascual-Diaz et al., 2021).

The plastomes of *Lactuca* and AE *Lactuca* species (one sample per species) were used for variation analysis and boundary comparisons. The intergenic spacer regions, intron sequences, and genes (such as *accD*, *matK*, *ndhF*, *rpl22*, and *ycf1*) with high variation and parsimoniously informative sites could be used for further common DNA barcode selection and fast identification of *Lactuca* and Lactucinae species (Amar, 2020; Meena et al., 2020; Li et al., 2021; Song et al., 2022; Tang et al., 2022). The junction sites of LSC, SSC, and IRs of *Lactuca* and AE *Lactuca* were conserved. Reverse SSC regions in chloroplast genomes were detected in 30 *Lactuca* and related species (34 taxa), especially in AE *Lactuca* species (13 taxa). It has been demonstrated that plastome has two equimolar states that differ in the relative orientation of SSC regions (Palmer, 1983). Reverse SSC regions were identified in other Asteraceae plastome studies as well, such as cultivated lettuce, sunflower (*Helianthus annuus* L.), and *Guizotia abyssinica* Cass. (Timme et al., 2007; Dempewolf et al., 2010). It has been pointed out that heteroplasmy in SSC orientation of plastomes within individual plants should not be overlooked in phylogenetic analyses (Walker et al., 2015). In this study, isomer inversions were also observed in two different individuals of one species (e.g., *L. saligna* and *L. georgica*). Therefore, reverse SSC orientations among lineages could be considered as natural variations within and between individuals instead of phylogenetic signals.

There are two common pseudogenes (*ψ rps19* and *ψ ycf1*) caused by the incomplete DNA fragments located on the IR boundaries of chloroplast genomes of *Lactuca* and AE *Lactuca*, which had been widely found in Asteraceae plastome studies (Wang et al., 2021). Another two common pseudogenes, *ψ ndhF* and *ψ psaJ*, were shared by certain related taxa. The pseudogenization of *ψ ndhF* was detected in 23 *Lactuca* taxa in Clades I and II, in which most of the species were considered as lettuce gene pool species (Koopman et al., 1998; Lebeda et al., 2007; van Treuren et al., 2012). Plastid *NDH* gene loss or pseudogenization has been reported in many angiosperms (Lin et al., 2017; Fu et al., 2021; Li et al., 2021). The pseudogenization of *ψ psaJ* occurred in four *Lactuca* and 13 AE *Lactuca* samples as results of early termination of genes. Photosystem I complex is composed of protein encoded by multiple chloroplast genes, including *psaJ*, *psaA*, *psaB*, *psaC*, and *psaI*, and nuclear genes (Varotto et al., 2002). Previous studies found that the growth rate and assimilation ability of *psaJ*-knockout mutants by chloroplast transformation in tobacco (*Nicotiana tabacum*) remained unchanged and were inhibited only under limited light intensity (Schottler et al., 2007). Pseudogenization of *ψ ndhF* and *ψ psaJ* in *Lactuca* and AE *Lactuca* does not seem to affect their growth significantly. A possible reason could be that functional copies of the genes have been transferred into the mitochondrial or nuclear genomes (Lin et al., 2015; Daniell et al., 2016).

The positive selection of Clade I and Clade II species (including *L. sativa*, *L. altaica*, *L. aculeata*, *L. serriola*, *L. georgica*, *L. scarioloides*, *L. virosa*, *L. saligna*, *L. viminea*, *L. reviersii*, *L. tetrantha*, and *L. orientalis*) was detected in five plastid-coding genes, *accD*, *atpF*, *cemA*, *clpP*, and *rpl22*.

In general, the plastid genes under positive selection of lettuce gene pool species were found to be involved in the processes of leaf and chloroplast development, photosynthesis, protein synthesis, fatty acid synthesis, other secondary metabolite synthesis, and protein differentiation and degradation, as well as energy metabolism (Hudson and Mason, 1988; Katoh et al., 1996; Majeran et al., 2000; Kode et al., 2005; Bock, 2007; Fleischmann et al., 2011; Olinares et al., 2011; Rodriguez-Concepcion et al., 2019; Caroca et al., 2021).

### 4.2 The delimitation among *Lactuca* and other lineages of lactucinae

The concept of *Lactuca* has been revised multiple times during the last century. The global distribution of *Lactuca* species was summarized and classified into four geographic groups (European, Asian, African, and American) (Lebeda et al., 2004b; Lebeda et al., 2019). Using ITS and five plastid markers, the molecular phylogeny of Lactucinae focusing on the Chinese lineages identified six lineages (*Cicerbita*, *Cicerbita II*, *Lactuca*, *Melanoseris*, *Notoseris*, and *Paraprenanthes*) as a monophyletic core group within the subtribe, and *Melanoseris* and *Lactuca* were inferred as sister groups (Wang et al., 2013). A new genus of Lactucinae, *Astartoseris*, was proposed as monospecific, and *L. triquetra* was designated as the type species (Kilian et al., 2017a). The contradictory systematic positions of *Astartoseris* (*L*. *triquetra*), *Faberia*, and *Prenanthes* inferred from nuclear and plastid phylogenetics were observed and discussed in previous studies, and eventually they were retained in Lactucinae based on morphology, karyology, and potential origins discussed in Wang et al. (2013); Kilian et al. (2017a), and Kilian et al. (2017b). Therefore, the three lineages were not discussed further in this work.

The molecular phylogeny using nrDNA, plastid DNA regions, and plastomes of *Lactuca*, focusing on the AE *Lactuca* species, recognized *Lactuca* as a polyphyletic group (Wei, 2016; Wei et al., 2017). Although the supporting values were not very high, the results indicated that the AE *Lactuca* is very likely to be a sister to *Melanoseris*. Kilian et al. (2017b) studied the diversification of the Lactucinae (Compositae: Cichorieae) and referred to the “*Cicerbita II* lineage” as a new genus “*Kovalevskiella*.” All of the lineages of Lactucinae were recognized until then, although the phylogenetic relationships among *Lactuca*, AE *Lactuca*, *Melanoseris*, *Notoseris*, and *Paraprenanthes* were not well resolved due to weak support values.

#### 4.2.1 The phylogenetic relationships among Lactucinae lineages

Compared to non-coding plastid and nrDNA regions, complete plastomes have been shown to be valuable and robust for phylogeny reconstruction of angiosperm groups (Zhao et al., 2020; Su et al., 2021; Wen et al., 2021). The complete plastome sequences in this study illustrated that the core *Lactuca* lineage is monophyletic with robust support. *Cicerbita* species are sisters to all of the other lineages, including *L. alatipes* + *Notoseris* + *Paraprenanthes*, *Kovalevskiella*, and *L. crambifolia* + *M*. *decipiens* + AE *Lactuca* + core *Lactuca* lineages. This study is the first molecular sampling of *L. alatipes*, and the results indicated that this species is very likely to be *Notoseris* or *Paraprenanthes*. *L. alatipes* was described to have a glabrous stem, basal leaves hastate, margin sinuate, and more rarely lyrately pinnate with one pair of small ovate lateral lobes; paniculiform, with few to many capitula; capitula with nine florets; achene reddish, narrow, very compressed, prominently 4–5 ribs on each side (Collett and Hemsley, 1890). We downloaded plastid DNA markers from a previous publication (Wang et al., 2013) and reconstructed phylogenetic analysis of *L. alatipes*, *Notoseris*, and *Paraprenanthes* species (**Supplementary Figure S4**). *L. parishii* LAC 028 in Wang et al. (2013) was identified as *P. umbrosa*, but later corrected as *P. parishii* LAC 028 in Kilian et al. (2017b). The results of phylogenetic analysis showed that *L. alatipes* should be transferred from *Lactuca* to *Paraprenanthes*. *L. alatipes* (type from 1890) is conspecific to *L. parishii* (type from 1911), and both names should be synonymized under *P*. *alatipes*.

Our samples included two species of *Kovalevskiella* lineage endemic to the Pamiro-Alai and western Tian Shan mountain ranges. They were discussed in Kilian et al. (2017b); there will be no more discussion about them in this work.

#### 4.2.2 African endemic *Lactuca* species

The plastome phylogeny revealed that *Lactuca* currently circumscribed (when AE is included) is polyphyletic. AE *Lactuca* is paraphyletic with respect to *Melanoseris* species (*M. decipiens* and *L. crambifolia*). The taxonomic position of *L. crambifolia* was identified and discussed in the study of SW Asian-centered Lactucinae species and thus is not further discussed here (Güzel et al., 2021).

The MRCA of the AE *Lactuca* species and the core group of “true” *Lactuca* species diverged around 7.14 (6.55–7.73) Mya, and the MRCA of AE *Lactuca* Clade diverged about 5.67 Mya, and the 95% HPD interval of the node age was 5.16–6.24 Mya. All of the species in this clade are distributed in Africa, while the MRCA of the core *Lactuca* species diverged early in Asia around 6.82 (6.27–7.40) Mya.

Despite the geographic distribution and evolutionary history, AE *Lactuca* species also have distinctive morphological characteristics from *Lactuca* species: 1) the achene is usually broadly thickened at the margins, 1–3 (rarely 3–6–7) rounded longitudinal ribs on each side, sometimes with furrows (Stebbins, 1937; Jeffrey, 1966; Dethier, 1982; Kilian, 2001; Wei et al., 2022), but *Lactuca* achenes have no obvious thickened margins or prominent rounded ribs on each side; 2) AE *Lactuca* mostly have 3(4)–5 florets per capitulum, seldom 9–10 or 10–14 or ±20 florets, whereas *Lactuca* have a variable number of florets, usually 10–30 florets, sometimes 5 or 5–10 florets (Kilian et al., 2009).

Although the systematic positions of AE *Lactuca* species and *Melanoseris* species inferred from plastomes and ITS sequences were discovered close, no apparent morphological discontinuity between the two groups was observed on important taxonomic features, such as floret number per capitulum, floret color, or chromosome number. *Lactuca attenuata* Stebbins (2n = 32), *L. glandulifera* (2n = 16), and *Lactuca homblei* De Wild. (2n = 16) are the only three known AE *Lactuca* species with chromosome number, while *Melanoseris* species are generally diploid (2n = 16) and rarely tetraploid (Kilian et al., 2009; Shih and Kilian, 2011).

As discussed above, AE *Lactuca* species have different divergence times and distinctive features from the core *Lactuca* lineage and thus should be excluded from *Lactuca*. To well resolve phylogenetic relationships between AE *Lactuca* and *Melanoseris*, more taxon sampling, especially Asian *Melanoseris*, should be taken into account for high-throughput sequencing and further taxonomic revisions.

### 4.3 The origin, phylogeny, and historical biogeography of *Lactuca* and the main phylogenetic ingroups

#### 4.3.1 The origin of *Lactuca*

The MRCA of *Lactuca* most likely originated in Asia-Temperate W and diverged around 6.82 Mya. This time estimation is younger than previous research using plastid DNA regions (Kilian et al., 2017b), in which the *Lactuca* taxa were not monophyletic and mixed with some *Melanoseris* taxa into one clade. The *Lactuca* lineages can be divided into nine ingroups, Clades I–IX. The MRCA of *Lactuca* species in Clade VIII and IX diverged around 5.89 Mya, and the *Lactuca* species in these two clades are now in America, Europe, Africa + Macaronesia, Asia-Temperate W, and Indian Subcontinent. The MRCA of *Lactuca* species in Clades I–VII was from Asia-Temperate W, and its progenies are now in Asia, Europe, Africa, and widely widespread, such as *L. serriola*, *L. altaica*, *L. virosa*, *L. saligna*, and *L. indica*.

#### 4.3.2 Phylogeny of *Lactuca*

In this section, we will discuss the phylogenetic relationships within *Lactuca* lineages starting from the basal clades of the phylogenetic tree to the tips.

**Clade IX.** The MRCA of this clade diverged around 3.09 Mya. This clade (2n = 16) includes *Lactuca palmensis* Bolle, an endemic perennial herb from La Palma in the Canary Islands (Dias et al., 2018), *Lactuca inermis* Forssk., a perennial herb from Tropical Africa to SW Asia, *L. tenerrima* Pourr. from Western Mediterranean to NW Africa, and *Lactuca picridiformis* Boiss from Afghanistan, Iran, and Pakistan. The systematic position of *L. picridiformis* based on plastome and ITS results was inconsistent. The chromosome number and morphology of the species suggest that plastid phylogenetic results are more reliable, and the ITS results might be gene introgression caused by hybridization (Kilian et al., 2017b).

**Clade VIII.** The MRCA of the NAE *Lactuca* (allopolyploid, 2n = 34, excluding *L. oblongifolia*) and *L. plumieri* (2n = 16) differentiated around 3.49 Mya. Thereafter, the MRCA of NAE *Lactuca* diverged very recently during the late Pleistocene (0.37 Mya) with high support values, much later than that in a previous study (Kilian et al., 2017b). *Lactuca watsoniana* Trel. (2n = 34) is endemic to the Azores in Macaronesia (Watson, 1870) and systematically related to the NAE *Lactuca* in our ITS trees. The North American-Azorean clade was also resolved as a sister to SW European Alpine-Pyrenean *L. plumieri* in previous studies (Dias et al., 2018; Jones et al., 2018).

NAE *Lactuca* in Clade VIII was considered to be derived from ancestral hybridization events between species with basic chromosome numbers x = 8 and x = 9 (Babcock et al., 1937). The potential progenitors of the allopolyploid *Lactuca* with a basic chromosome number x = 8 could be *L. plumieri* (or its extinct ancestors), and x = 9 progenitors might be *L. tatarica*, *L. quercina*, *L. sibirica*, or *L. indica* (or their extinct ancestors of clade) (Jones et al., 2018). The morphological characteristics of florets and achenes among NAE *Lactuca* lineages, *L. plumieri*, *L. sibirica*, *L. tatarica* (Jones et al., 2018), and *L. indica* (Jones et al., 2018; Wei et al., 2022) also indicated similarities and possible hybridization events.

The MRCA of NAE *Lactuca* (excluding *L. oblongifolia*) and *L. plumieri* diverged around 5.89 Mya, very close to the divergence time (5.88 Mya) of MRCA of Clade III (including *L. quercina*, *L. sibirica*, *L. tatarica*, and *L. oblongifolia*), Clade IV (including *L. indica*), and Clade V. Just as Jones et al. (2018) proposed, the MRCAs of these two groups were likely to be once widespread concurrently from Europe, Africa, and Asia and migrated to America *via* North Atlantic land bridges (NALBs) (x = 8 progenitors) and Bering land bridges (BLBs) (x = 9 progenitors). The MRCAs of these two groups hybridized to produce x = 17 lineages in America, and then the diploid progenitors became extinct in America in late Pliocene, resulting in the contemporary geographic isolations of x = 8 and x = 9 *Lactuca* from NAE *Lactuca*.

**Clade VII.** The MRCA of *L. glauciifolia*-*L. intricata* clade (2n = 18) diverged from its sister Clades I–VI around 6.75 Mya, and all of the species shared a common *ψ clpP.* The species in this clade are now mainly distributed in the E Mediterranean and SW Asia and extend widely to Europe and Central Asia. Although the systematic positions of *L. picridiformis*, inferred plastome, and ITS data were contradicting, the plastome tree with high confidence values was supported by karyology and morphological characteristics (Kilian et al., 2009; Kilian et al., 2017b).

**Clade VI.** This clade includes perennial herbaceous species. The MRCA of Clade VI diverged from its sister Clades III–V around 6.12 Mya and originated from Asia-Temperate W and subsequently spread to Asia-Temperate E, Indian Subcontinent, and Asia-Tropical. Clade VI can be divided into two subclades, VI-1 and VI-2. Clade VI-1 includes Turkish endemic *Lactuca* species (*Lactuca boissieri* Rouy, *L. variabilis*, and *Lactuca glareosa* Schott and Kotschy), *L. mulgedioides* (distributed only in Syria, Lebanon, and Turkey) (Plastome data), and *Lactuca fenzlii* N.Kilian and Greuter endemic to Turkey, *Lactuca aurea* (Sch.Bip. ex Vis. and Pančić) Stebbins from Europe, and *Lactuca deltoidea* DC. ex C.A.Mey. near Caucasus (present in ITS trees). Clade VI-2 contains *L. dissecta* from SW to Central Asia and *L. dolichophylla* from SW Asia to Sino-Himalaya. The species composition of the clade was consistent with previous molecular and morphological results (Kilian et al., 2017b; Güzel et al., 2021).

**Clade V.** The MRCA of Clades III–V diverged around 5.88 Mya and originated from the Asia-Temperate W and then diffused globally. Clade V contains only *L. adenophora* and *L. racemosa* (2n = 16) in plastome trees, and these two species plus *Lactuca macrophylla* A. Gray present in ITS trees. *L. macrophylla* is morphologically similar to *L. racemosa* and *L. adenophora* and was reported to have some sterile individuals, indicating possible ancient reticulation, chloroplast capture, or introgressive hybridization among these species (Güzel et al., 2018; Güzel et al., 2021). Additionally, the achenes of *L. macrophylla* and *L. racemosa* are apically attenuate, very rare for *Lactuca* lineages, whereas the achene of *L. adenophora* is beaked (Güzel et al., 2018). The systematic positions of species in Clade V were both resolved within *Lactuca* lineages in our plastome and ITS trees with different sister clades. In Güzel et al. (2021), Clade V was part of *Lactuca* in ITS tree, but one of the Asian *Melanoseris* clades (not included in this study) was resolved as its sister group in the plastid tree. Therefore, these species should be retained in *Lactuca*.

**Clade IV.** This Clade includes species mainly in Asia-Temperate E, namely, *L. indica*, *Lactuca raddeana* Maxim., *Lactuca formosana* Maxim., and *Lactuca triangulata* Maxim. (2n = 18). The MRCA of Clades IV and III diverged in Asia Temperate regions about 5.65 Mya and spread to Central Asia and NE Asia. Jones et al. (2018) treated *Lactuca jamaicensis* Griseb, native to Jamaica, as a synonym of *L. indica* and considered its distribution as a consequence of human introduction. All of the species in Clade IV were once established as an independent genus *Pterocypsela* (Shih, 1988). The main characteristics of *Pterocypsela* are capitulum with 9–25 yellow florets, achenes black, ellipsoid and compressed, and broadly winged at the edges (Shih, 1988; Wei et al., 2022). Molecular evidence, including our plastome data, showed that this genus should be retained in *Lactuca* (Wang et al., 2013; Kilian et al., 2017b; Wei et al., 2017). Hybridization experiments showed that *L. indica*, *L. raddeana*, and *L. tatarica* were closely related and could produce sterile progeny (Thompson et al., 1941; Whitaker and Thompson, 1941).

**Clade III.** This clade includes *L. bourgaei* (2n = 16), *L. quercina* (2n = 18), and three closely related perennial herbaceous plants *L. sibirica*, *L. tatarica*, and *L. oblongifolia* (2n = 18). The phylogenetic relationships of these species are closely related in plastome data but dissected into three clades in nuclear data. Clade III-1 is *L. quercina*, closely related to the crop lettuce and its relatives (Clade I) in nuclear data but a sister of *L. sibirica*, *L. tatarica*, and *L. oblongifolia* in the plastome trees. *L. quercina* is different from *L. sibirica*, *L. tatarica*, and *L. oblongifolia* in the characteristics of lingual floret color and achenes: *L. quercina* inflorescences with 16–22 yellow florets, achenes base truncate, with completely annular carpopodium, corpus attenuate into a beak, beak 1.5–3.9 mm long, oblong-ellipsoid and compressed, black with 5–6 prominent ribs on each face (Güzel et al., 2018); the inflorescences of *L. sibirica*, *L. tatarica*, and *L. oblongifolia* generally have about 20 purple, blue, or nearly white florets, achenes narrowly ellipsoid, flattened and subcompressed, apically attenuate or with a ca. 1–2 mm beak, 4–5 or 5–7 narrow ribs on either side (Shih and Kilian, 2011). Clade III-2 contains *L. oblongifolia*, *L. sibirica*, and *L. tatarica* and is a sister of Clade II in ITS trees. *L. sibirica* and *L. tatarica* could produce fertile hybrid progeny (Koopman et al., 2001). The incongruence of relationships among *L. sibirica*, *L. tatarica*, and *L. oblongifolia* between plastomes and ITS sequences may reflect hybridization and introgression between *L. sibirica* and *L. tatarica*. Clade III-3 is *L. bourgaei* within the *Melanoseris* Clade in ITS trees. A previous study demonstrated the compatibility relationship between *L. tatarica* and *L. bourgaei* and produced sterile hybrids (Thompson, 1943). Therefore, this study retained *L. bourgaei* in *Lactuca*. Further studies with more molecular and genetic evidence are needed in the future.

**Clade II.** This clade is a sister of Clade I, and their MRCA originated from Asia-Temperate W and diverged around 5.20 Mya. Clade II contains four species (2n = 18): *L. viminea*, *L. reviersii*, *L. tetrantha*, and *L. orientalis*, as well as additional *Lactuca acanthifolia* Benth. and Hook.f. and *Lactuca alpestris* (Gand.) Rech.f. in the ITS trees. These species are natively distributed in Europe, N Africa + Macaronesia, Asia-Temperate, and Indian Subcontinent (Feráková and Májovský, 1977; Bano, 2009; Kilian et al., 2009; Bano and Qaiser, 2011). Some species are narrowly distributed, such as *L. reviersii* endemic to Morocco in NW Africa, *L. tetrantha* endemic to Cyprus (east Mediterranean island), *L. acanthifolia* endemic to Turkey and Greece, and *L. alpestris* endemic to Kriti island in Greece (https://portal.cybertaxonomy.org/flora-greece/intro).

**Clade I.** The MRCA of Clade I originated from Asia-Temperate W region and diverged around 2.58 Mya, and all of the species in the clade (excluding unknown *L. scarioloides*) have a chromosome number of 2n = 18. A recent whole-genome resequencing of 445 *Lactuca* accessions revealed that cultivated lettuce was domesticated near the Caucasus (Wei et al., 2021), included in our geographic region of Asia-Temperate W (F). Clade I contains the important economic vegetable cultivated lettuce (*L. sativa*) and lettuce gene pool species (Koopman et al., 1998). The primary lettuce gene pool includes *L. aculeata*, *L. altaica*, *Lactuca azerbaijanica* Rech. f., *L. scarioloides*, and *L. serriola*. The secondary gene pool includes *L. saligna*. The tertiary gene pool contains 13 wild *Lactuca* species, namely, *L. acanthifolia*., *L. alpestrias*, *L. aurea*, *L. georgica*, *Lactuca longidentata* Moris ex DC., *L. orientalis*, *L. quercina*, *L. sibirica*, *L. tatarica*, *Lactuca taraxacifolia* (Willd.) Schumach., *L. viminea*, *L. virosa*, and *L. watsoniana* (Zohary, 1991; Koopman et al., 1998; van Treuren et al., 2012; Wei et al., 2017; Wei et al., 2021).

There are three subclades in Clade I. The *L. saligna* clade is the sister of the other two clades, *L. scarioloides*-*L. georgica*-*L. virosa* clade and *L. sativa*-*L. serriola*-*L. aculeata*-*L. altaica* clade. The systematic positions of *L. saligna* were congruent between plastome and ITS trees. A previous study revealed similar mitochondrial genome sequences and structures between *L. sativa* and *L. serriola*, but that of *L. saligna* was shown to be significantly different (Kozik et al., 2019). *L. saligna* was placed between *L. georgica*-*L. virosa* clade and *L. sativa*-*L. serriola*-*L. aculeata*-*L. altaica* clade in the most recent coalescence-based phylogenetic tree inferred from 4,513 single-locus nuclear genes (Wei et al., 2021). *L. saligna* is a non-host for lettuce downy mildew (*Bremia lactucae*) and can hybridize with the crop lettuce *L. sativa* for improvement of genetic resistance to downy mildew (Lebeda and Reinink, 1994; Jeuken et al., 2001; Jeuken and Lindhout, 2002; Zhang et al., 2009). Therefore, *L. saligna* is genetically more closely related to cultivated lettuce and should be retained as the second gene pool species.

The phylogenetic relationship between *L. georgica* and *L. virosa* is very close, and they are morphologically similar: *L. georgica* is a perennial with solitary glabrous stems, capitula cylindrical with about 15 small florets, yellow corolla, achenes broadly obovoid, dark purple or nearly black, slightly flat or flattened, narrowly winged, 6–7 mm long, with a light or dark brown filamentous beak of about 2–2.5 mm, with usually seven distinct ribs on each side, distributed mainly in Asia-Temperate W; the latter has numerous heads with about 15 yellow ligulate florets, achenes broadly elliptical, narrowly winged, about 6–10 mm long, five ribs, distributed in S Europe and N Africa (Feráková and Májovský, 1977; Kilian et al., 2009; Beharav et al., 2018; Wei et al., 2022). The plastome of *L. scarioloides* is the first molecular sampling of the species, and the Irano-Turanian wild lettuce was reported to be morphologically similar to *L. serriola*, despite leaf shape and achene and capitula size (Zohary, 1991). The native distribution regions of *L. georgica* and *L. scarioloides* are overlapped, indicating a possible hybridization introgression or chloroplast capture between them, which may also explain the relationships among *L. scarioloides*, *L. georgica*, and *L. virosa* inferred from plastome data.

Another research based on mitochondrial genomes found that *L. sativa* ‘Salinas’ and *L. virosa* were more closely related than *L. sativa* ‘Wendel’ and *L. serriola* (Fertet et al., 2021), confirming the involvement of *L. virosa* in the breeding history of Salinas varieties (Mikel, 2007). The differences of the relationships among *L. saligna*, *L. georgica*, *L. virosa*, and *L. sativa*-*L. serriola*-*L. aculeata*-*L. altaica* clade, inferred from whole genomes and organelle (plastid and mitochondrion) genomes, are likely to be the consequences of the complex artificial domestication history of lettuce. In terms of hybridization introgression, the complete genome sequences of cultivated lettuce and its relatives are more reliable to reflect the true evolutionary history than organelle genomes. Thus, we think that *L. georgica*, *L. scarioloides*, *L. virosa*, and *L. quercina* (discussed in Clade III) should be placed in the tertiary gene pool.

The species in *L. sativa*-*L. serriola*-*L. aculeata*-*L. altaica* clade can cross with each other to produce progeny. These wild species belong to the primary gene pool of cultivated lettuce (Koopman et al., 1998; Koopman et al., 2001; Wei et al., 2021). Our plastome data showed that *L. sativa* and *L. aculeata* were closer than *L. serriola* and *L. altaica*, but the ITS tree indicated that *L. serriola* and *L. altaica* were closer to *L. sativa* than *L. aculeata*, consistent with genome sequences (Wei et al., 2021). Previous studies treated *L. altaica* and *L. serriola* as conspecific species (Koopman et al., 1998; Koopman et al., 2001; Wei et al., 2017), but the recent whole-gene sequencing study showed that they were two independent species with different morphological characteristics (Wei et al., 2021). *L. altaica* usually has 7 (9) to 15 (17) florets ligulate, achenes light brown oblanceolate compressed, 3.5 mm long and 1 mm wide, with 6–8 ribs on each side, apex acute filamentous beak 3 mm long. *L. serriola* has about (7) 10–30 (50) small florets, achenes oblong-ovate, brown or olive-gray to grayish, achene body 3.0–4.0 mm long and 1.2–1.5 mm wide, with 5–8 ribs on each side, beak white, filiform, 3.0–4.5 mm long (Feráková and Májovský, 1977; Shih, 1997; Shih and Kilian, 2011). *L. azerbaijanica* has not yet been studied using molecular evidence and is retained temporarily as a primary lettuce gene pool species.

The remaining species, closely related to lettuce but not reported to be compatible to *L. sativa*, were treated as an extension of the tertiary gene pool. *L. indica* has been cultivated for its edible leaves (Kadereit and Jeffrey, 2007) and also has some resistance to lettuce downy mildew (van Treuren et al., 2011). Hybridization experiments have shown that *L. indica* and *L. sativa* can produce fusiform brown achenes with strongly compressed body and filiform white beak. The fertility of F_1_ hybrid seedlings is ongoing being tested (unpublished data from Zhen Wei). Previous studies reported that somatic hybridization of *L. sativa* and *L. tatarica* produced fertile hybrids (Maisonneuve et al., 1995). Three wild species, *L. aurea* (2n = 16), *L. longidentata* (2n = 16), and *L. watsoniana* (2n = 34) have different chromosome numbers from *L. sativa* (2n = 18) (Feráková and Májovský, 1977; Dias et al., 2018) and therefore should be excluded from the lettuce gene pool. *L. taraxacifolia* has been renamed as *Lactuca alaica* Kovalevsk. and transferred to *Kovalevskiella* lineage (Kilian et al., 2017b). Considering phylogenetic positions, morphological features, and crossing experiments, we added *L. indica*, *L. oblongifolia*, *L. reviersii*, and *L. tetrantha* to the extension list of the tertiary gene pool. In total, 11 species in the extension list could be potential genetic resources for future lettuce breeding.

As we discussed above, the lettuce gene pool was modified as follows:

1) The primary gene pool: *L. aculeata, L. altaica, L. serriola*, and *L. azerbaijanica*
2) The secondary gene pool: *L. saligna*
3) The tertiary gene pool: *L. georgica, L. scarioloides*, and *L. virosa*
4) Extension of the tertiary gene pool: *L. acanthifolia, L. alpestris, L. indica, L. oblongifolia, L. orientalis, L. quercina, L. reviersii, L. sibirica, L. tatarica, L. tetrantha*, and *L. viminea*

### Taxonomic conclusion

*Paraprenanthes alatipes* (Collett and Hemsl.) Z. Wei & S.X. Zhu, comb. nov. ≡ *Lactuca alatipes* Collett & Hemsl. in J. Linn. Soc., Bot. 28: 79. 1890 – Type: Burma, Shan hills terai, 910 m, 1888, H. Collett 479.

= *Lactuca parishii* Craib in Kew Bull. 1911: 403. 1911, syn. nov. – Type: Burma, Moulmein, 1200-1500 m, 1862, Parish 423.

## Data availability statement

The datasets presented in this study can be found in the GenBank (https://www.ncbi.nlm.nih.gov/genbank/) under accession number ON782474-ON782569 (plastome data) and OP070064-OP070156 (ITS data).

## Author contributions

ZW, FTB and ES conceived the study. ZW conducted the experiments. RC, ZW, ZWL, HC, YGM and XMX analyzed the data. ZW and RC wrote the manuscript. FTB, ES, XMX, SXZ, ZWL, HC and YGM reviewed and edited the manuscript. SXZ did field investigation and material collection.

## Funding

This work was supported by the National Natural Science Foundation of China (31700175; 31670188); Key Research and Development Project of Henan Province (221111110800); Fostering Project for Young Teachers of Zhengzhou University (JC21343014; JC21343016); Qinghai Provincial Department of Science and Technology (2020-ZJ-Y04); SYNTHESYS Joint Research Activities 4 (JRA4: Plants/fungi herbarium DNA).

## Acknowledgments

We would like to thank all the curators, dr. ir. J.J. Wieringa (Naturalis Biodiversity Center, Leiden, The Netherlands), Dr. Alan Paton, Ms. Sally Dawson, and Ms. Marie Briggs (Royal Botanic Gardens, Kew, The United Kingdom), Ms. Suzanne Cubey (Royal Botanic Gardens, Edinburgh, The United Kingdom), Ms. Ranee Prakash (Natural History Museum, London, The United Kingdom), Dr. Xiaohua Jin (Institute of Botany, Chinese Academy of Sciences, China) for helping us obtain the herbarium samples.

## Conflict of interest

The authors declare that the research was conducted in the absence of any commercial or financial relationships that could be construed as a potential conflict of interest.

## Publisher’s note

All claims expressed in this article are solely those of the authors and do not necessarily represent those of their affiliated organizations, or those of the publisher, the editors and the reviewers. Any product that may be evaluated in this article, or claim that may be made by its manufacturer, is not guaranteed or endorsed by the publisher.
