# Supplementary figures and images for: Plastome-based phylogeny and biogeography of Lactuca L. (Asteraceae) support revised lettuce gene pool categories
Source: Front Plant Sci. 2022 Oct 12;13:978417. doi: 10.3389/fpls.2022.978417 (PMC9597326; doi:10.3389/fpls.2022.978417)

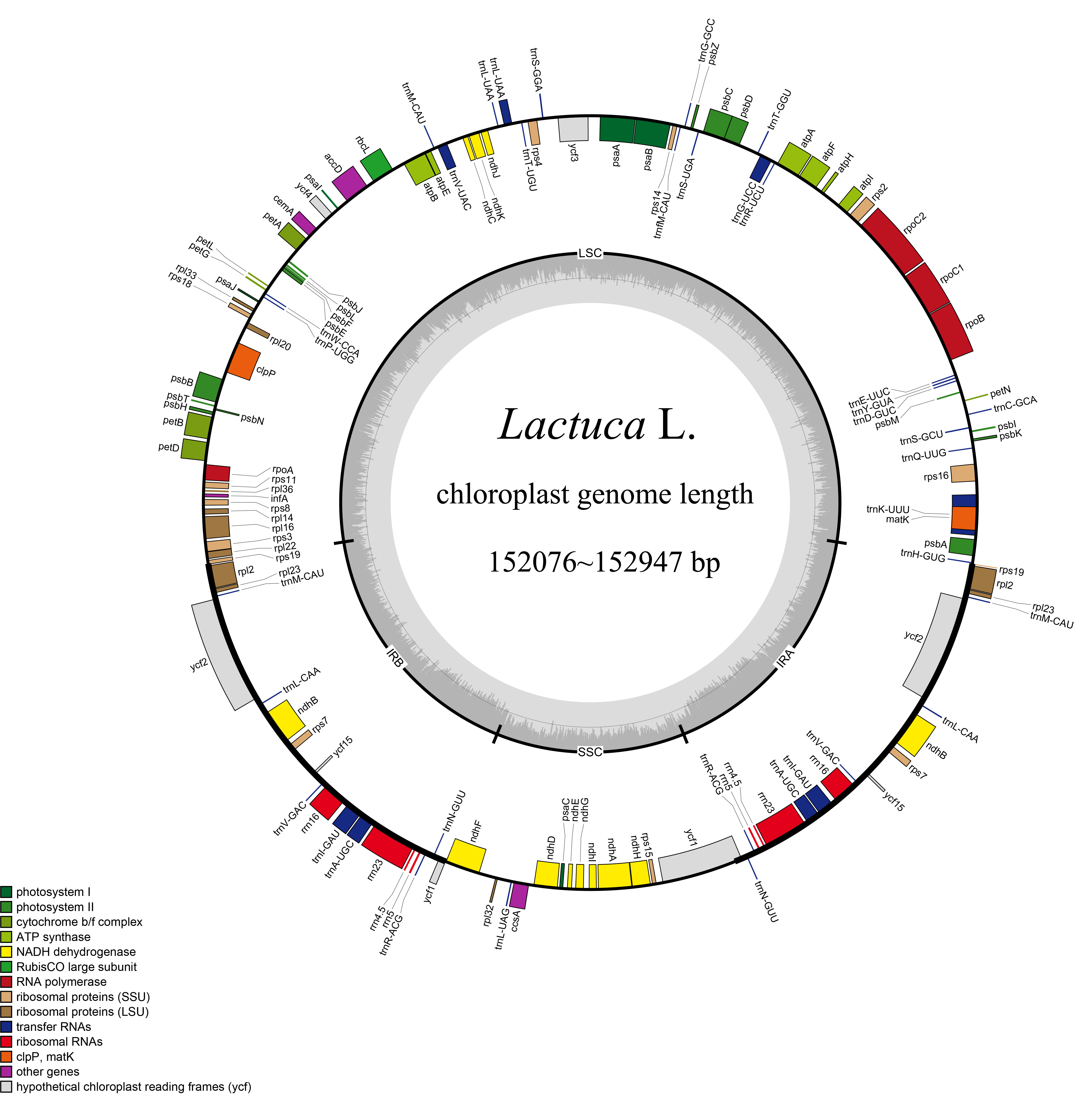

Supplement: Supplementary file 1 [file Image_1.jpeg]

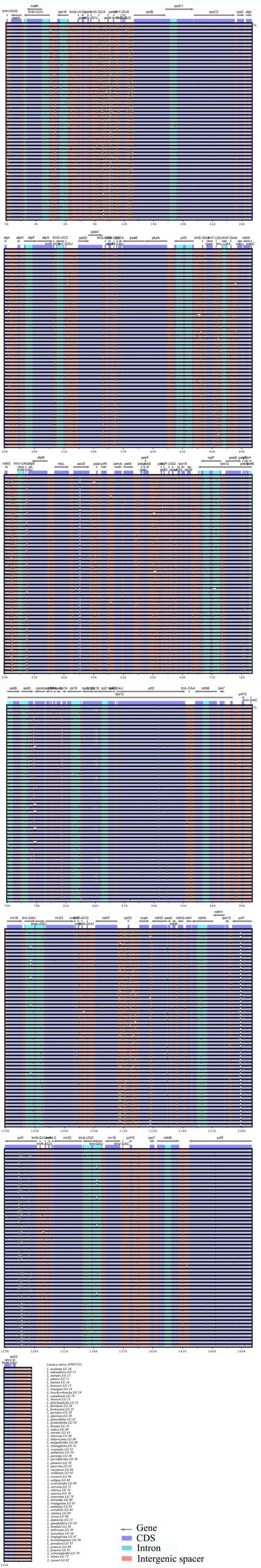

Supplement: Supplementary file 2 [file Image_2.jpeg]

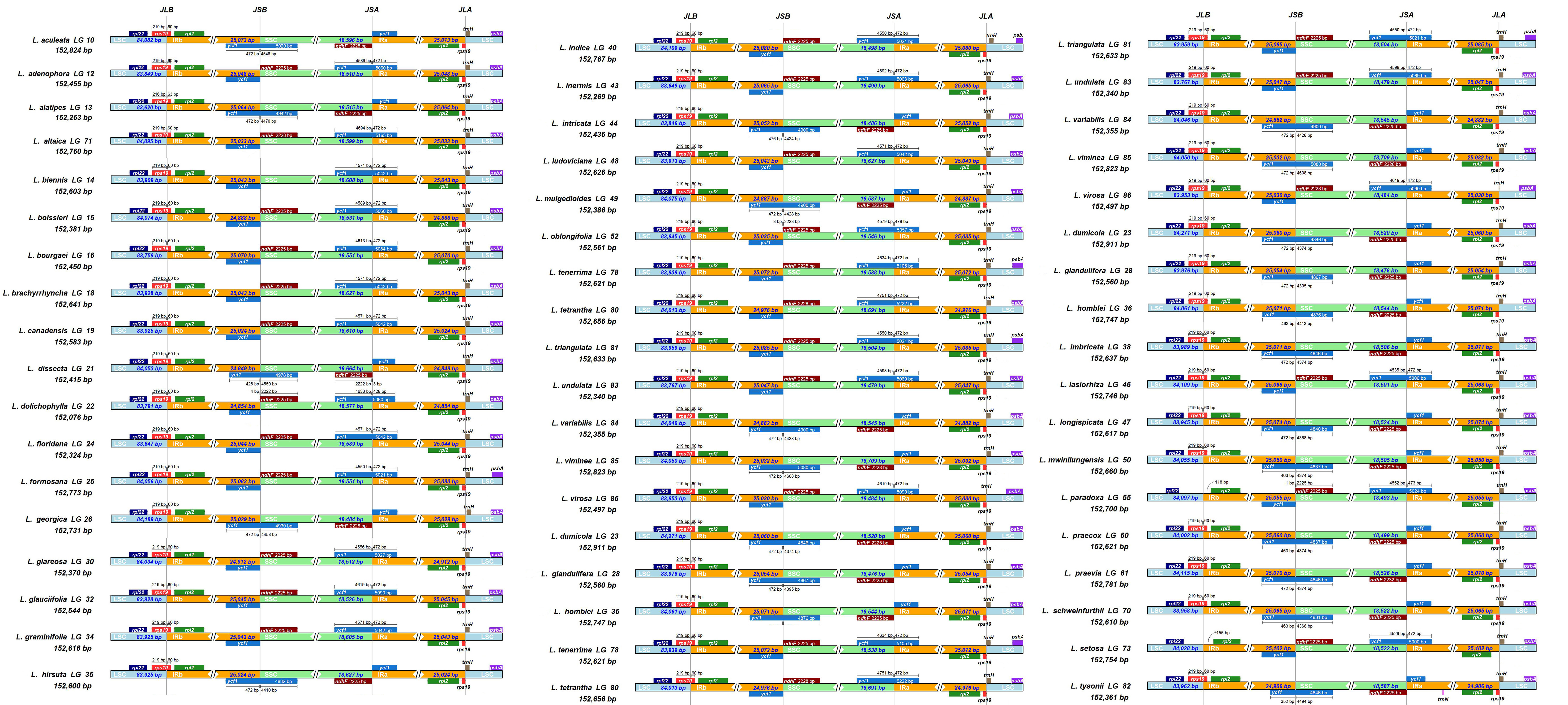

Supplement: Supplementary file 3 [file Image_3.jpeg]

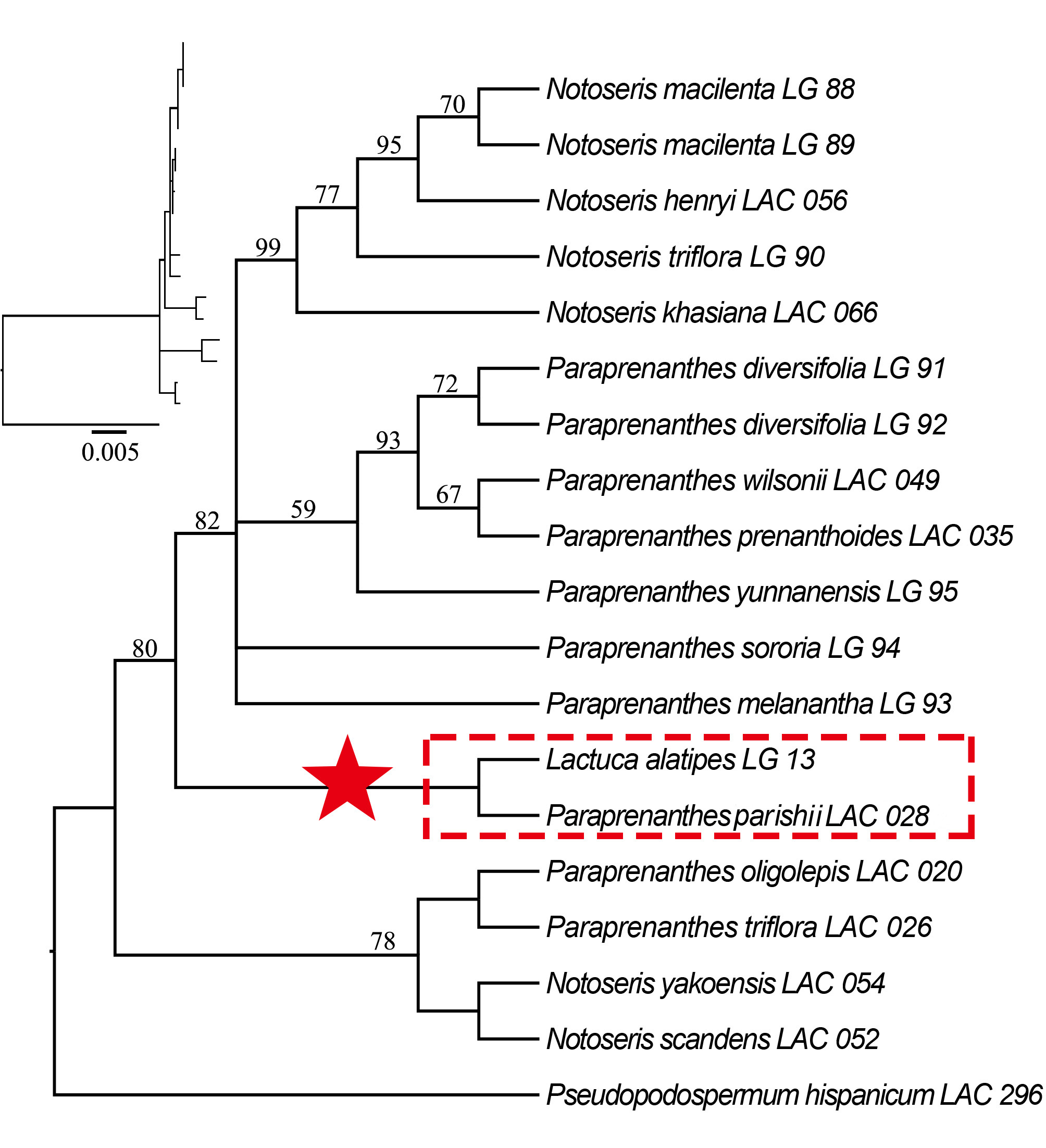

Supplement: Supplementary file 4 [file Image_4.jpeg]

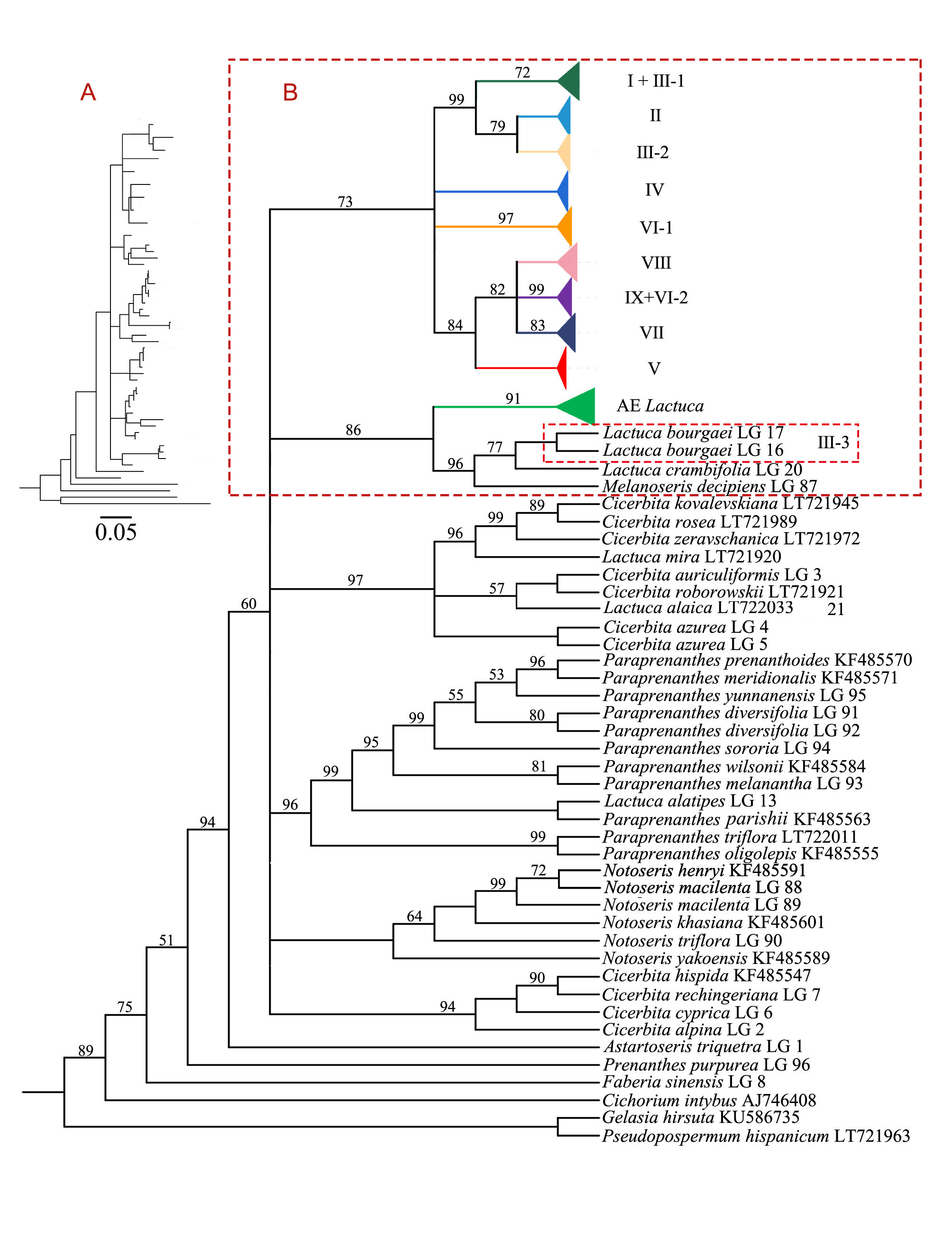

Supplement: Supplementary file 5 [file Image_5.jpeg]

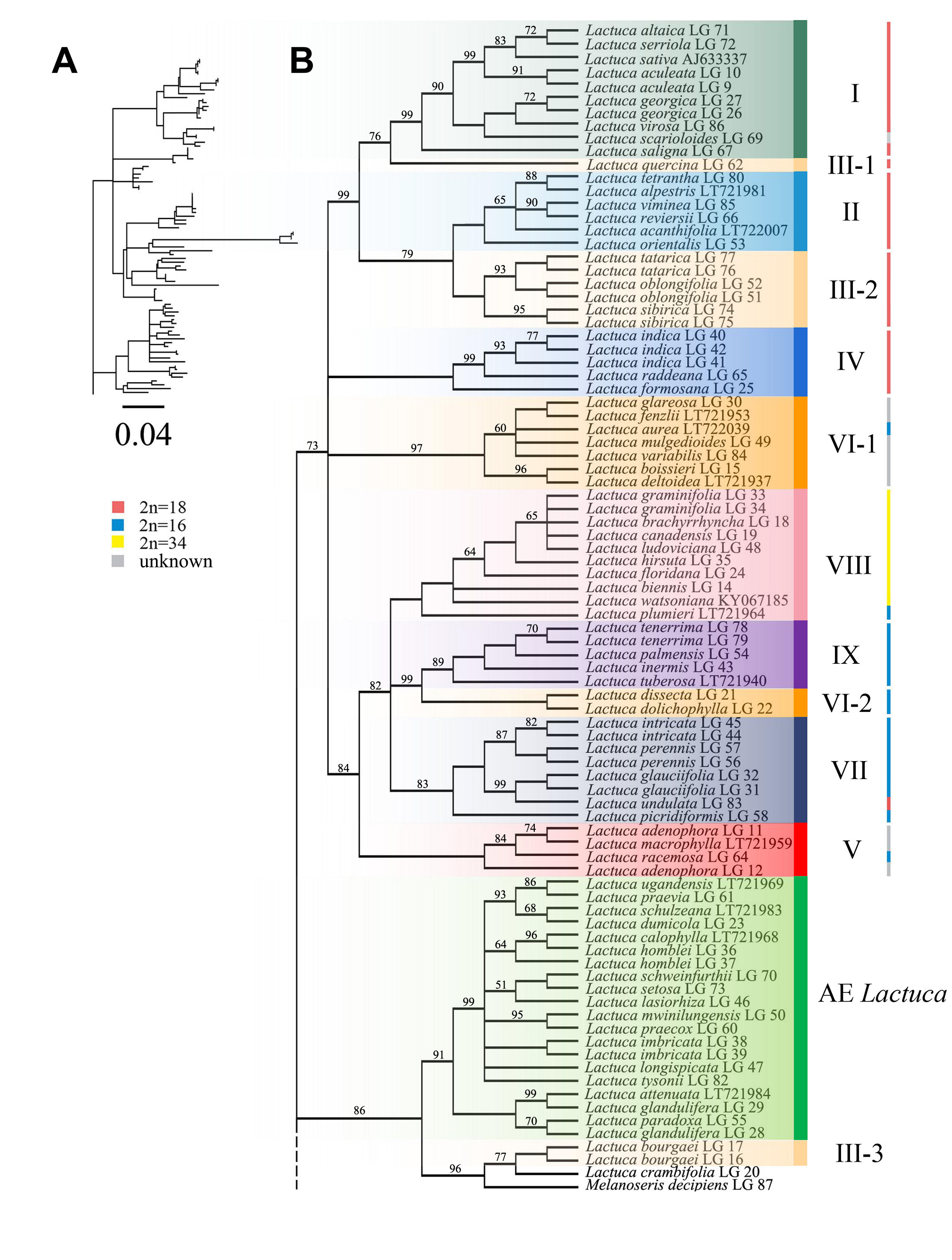

Supplement: Supplementary file 6 [file Image_6.jpeg]

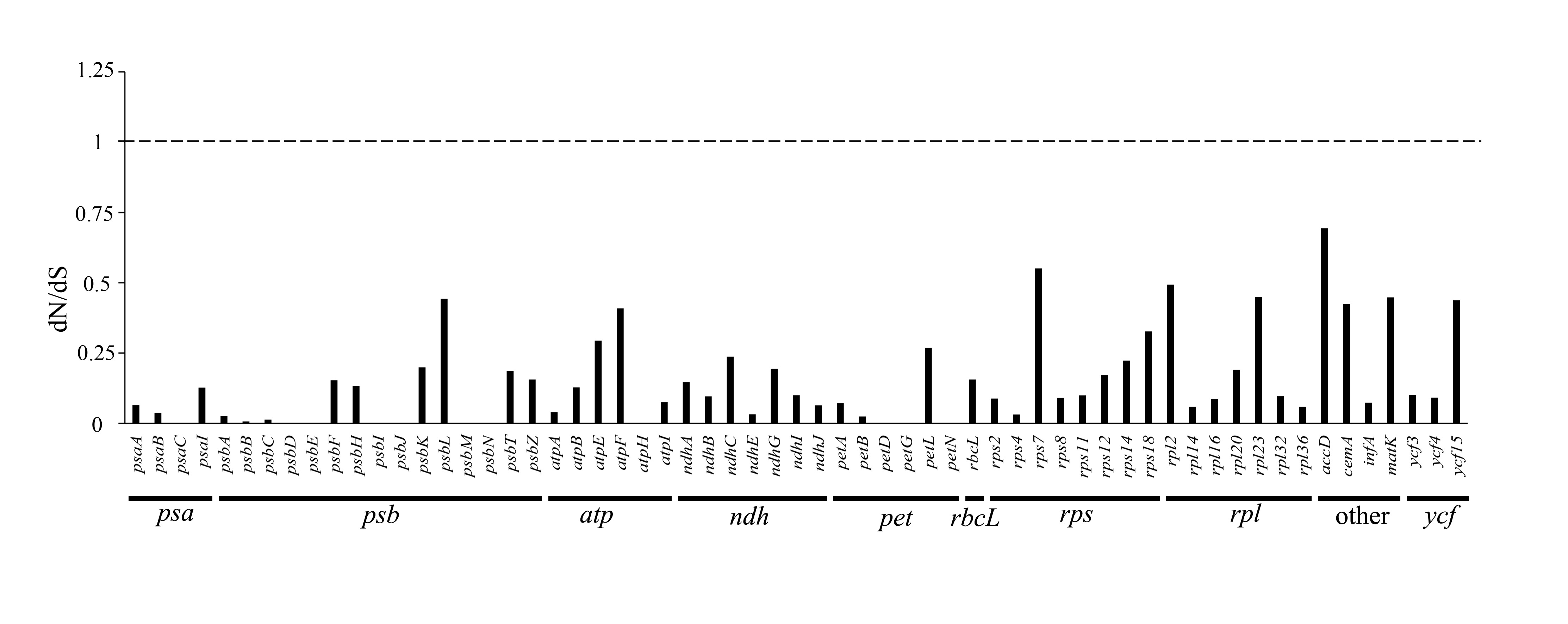

Supplement: Supplementary file 7 [file Image_7.jpeg]

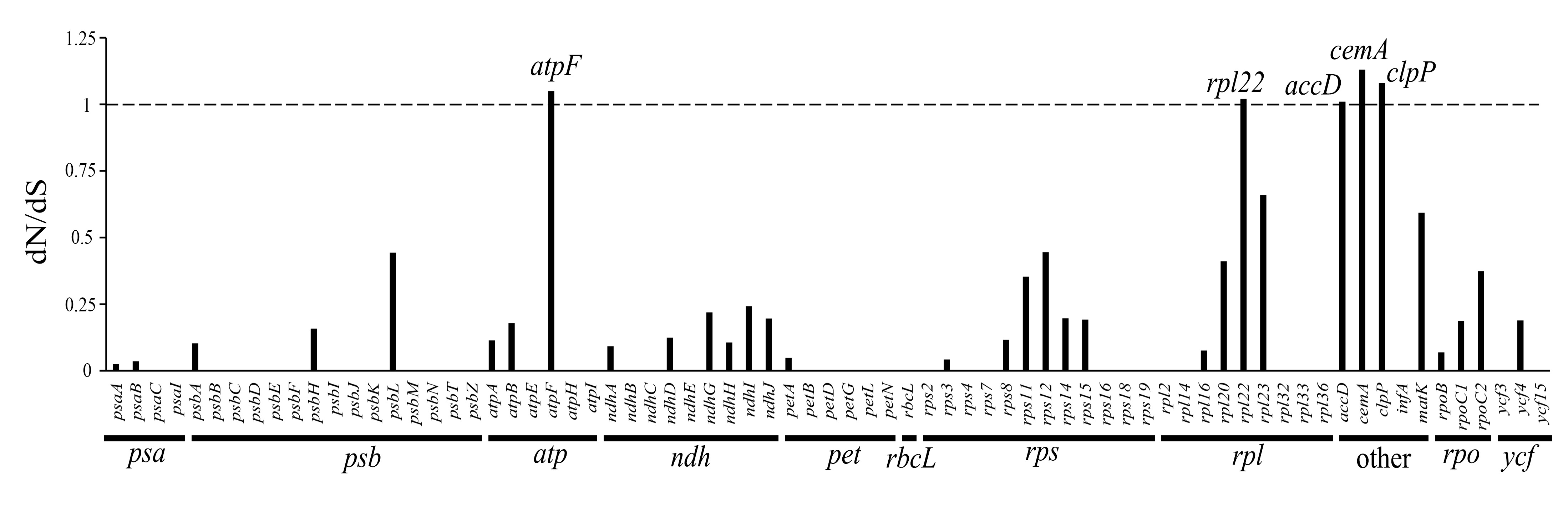

Supplement: Supplementary file 8 [file Image_8.jpeg]
