# Supplementary material for: Plastome-based phylogeny and biogeography of Lactuca L. (Asteraceae) support revised lettuce gene pool categories
Source: Front Plant Sci. 2022 Oct 12;13:978417. doi: 10.3389/fpls.2022.978417 (PMC9597326; doi:10.3389/fpls.2022.978417)
Supplement: Supplementary file 10 [file DataSheet_1.docx]

Supplementary Material

# Supplementary Figures and Tables

## Supplementary Tables

**Supplementary Table 1.** Information of plant materials.

| Sample No. | Species | Collector | Herbarium | Collector No. | Collection Date | Location | Illumina Sequencing platform |
| --- | --- | --- | --- | --- | --- | --- | --- |
| LG 1 | *Astartoseris triquetra* (Labill.) N.Kilian, Hand, Hadjik. | Trott, A.C. | K | 2045 | 1954 | Lebanon | HiSeq 4000 |
| LG 2 | *Cicerbita alpina* Wallr. | Breteler, F. J. | WAG | 7538 | 1977 | France | HiSeq 2000 |
| LG 3 | **Cicerbita auriculiformis* (C.Shih) N.Kilian | Sun, X. G. | PE | 6367 | 2006 | China | HiSeq Xten |
| LG 4 | **Cicerbita azurea* Beauverd | Zhu, S.X. & Gao, T. G. | PE | 112 | 2002 | China | HiSeq Xten |
| LG 5 | *C. azurea* | Chen, Y. L. | PE | 06-1 | 2006 | China | HiSeq Xten |
| LG 6 | *Cicerbita cyprica* (Rech.f.) M.Güzel, Coșkunç. & N.Kilian | Davis, P. H. | E | 3503 | 1941 | Cyprus | HiSeq 4000 |
| LG 7 | *Cicerbita rechingeriana* (Tuisl) Coșkunç., M.Güzel & N.Kilian | Wheeler, H. R. | E | 699 | 1956 | - | HiSeq 4000 |
| LG 8 | *Faberia sinensis* Hemsl. | Beijing Group | PE | 893093 | 1989 | China | HiSeq Xten |
| LG 9 | *Lactuca aculeata* Boiss. | Myaytop, T. | E | 23125 | 1972 | Turkey | HiSeq 4000 |
| LG 10 | *L. aculeata* | Koopman, W. J. M. | WAG | 15692 | 1995 | Turkey | HiSeq 2000 |
| LG 11 | *Lactuca adenophora* Boiss. & Kotschy | Greuter, W. R. | E | 12928 | 1975 | - | HiSeq 4000 |
| LG 12 | *L. adenophora* | Hamsa, H. E | E | 44924 | 1966 | Turkey | HiSeq 4000 |
| LG 13 | *Lactuca alatipes* Collett & Hemsl. | Herrst, J. | K | 10228 | 1926 | Thailand | HiSeq 4000 |
| LG 14 | *Lactuca biennis* (Moench) Fernald | Fernald, M. L. & Wieband, K. M. | BM | 6421 | 1911 | Canada | HiSeq 4000 |
| LG 15 | *Lactuca boissieri* Rouy | Güner, A. & Yildiz, B. | E | 4081 | 1981 | Turkey | HiSeq 4000 |
| LG 16 | *L. bourgaei* Irish & Taylor | Cultivated Plant of the RBGE | E | 693877 | 1973 | England | HiSeq 4000 |
| LG 17 | *L. bourgaei* | Davis & Hedge | E | 32465 | 1957 | Turkey | HiSeq 4000 |
| LG 18 | *Lactuca brachyrhyncha* Hayata | Pringle, C.G. | BM | 6883 | 1898 | Mexico | HiSeq 4000 |
| LG 19 | *Lactuca canadensis* L. | Paul B.M. et al. | PE | 3537 | 2005 | USA | HiSeq Xten |
| LG 20 | *Lactuca crambifolia* Boiss. | Hedge, I. C. & Wendelbo, P. | E | 3058 | 1962 | Afghanistan | HiSeq 4000 |
| LG 21 | *Lactuca dissecta* D.Don | Hedge, I., Wendelbo, P. & Ekberg, L. | E | 8619 | 1996 | Afghanistan | HiSeq 4000 |
| LG 22 | *Lactuca dolichophylla* Kitam. | Polunin, O. V. | E | 14810 | 1977 | India | HiSeq 4000 |
| LG 23 | *Lactuca dumicola* S.Moore | John G. | BM | 1728 | 1903 | Angola | HiSeq 4000 |
| LG 24 | *Lactuca floridana* (L.) Gaertn. | Fosbeg, F. R. | PE | 23936 | 1945 | USA | HiSeq Xten |
| LG 25 | *Lactuca formosana* Myaxim. | Zhu, S. X. | ZZU | 2011-1576 | 2011 | China | HiSeq 2000 |
| LG 26 | *Lactuca georgica* Grossh. | Akhani, H. | E | 11840 | 1995 | Iran | HiSeq 4000 |
| LG 27 | *L. georgica* | Purse, P. | K | 9020 | 1966 | Iran | HiSeq 4000 |
| LG 28 | *Lactuca glandulifera* Hook.f. | Newbould, J. & Harley, R. M. | K | 4633 | 1959 | Tanzania | HiSeq 4000 |
| LG 29 | *L. glandulifera* | Hilliard, O. M. & Burrt, B. L. | E | 6094 | 1971 | Malawi | HiSeq 4000 |
| LG 30 | *Lactuca glareosa* Schott & Kotschy | Darrah, J. | E | 398 | 1969 | Turkey | HiSeq 4000 |
| LG 31 | *Lactuca glauciifolia* Boiss. | Hedge, I., Wengelbo, P. & Ekberg, L. | E | 7775 | 1969 | Afghanistan | HiSeq 4000 |
| LG 32 | *L. glauciifolia* | Rechinger, K.H. | K | 29167 | 1965 | Pakistan | HiSeq 4000 |
| LG 33 | *Lactuca graminifolia* Michx. | Bozeman, J. R. & Logue J.F. | E | 9158 | 1967 | USA | HiSeq 4000 |
| LG 34 | *L. graminifolia* | Jr. Lane R.L. | BM | 2631 | - | Georgia | HiSeq 4000 |
| LG 35 | *Lactuca hirsuta* Muhl. ex Nutt. | Robbins, J. W. | K | - | 1865 | USA | HiSeq 4000 |
| LG 36 | *Lactuca homblei* De Wild. | Zimmer, F. | BM | - | - | Tanzania | HiSeq 4000 |
| LG 37 | *L. homblei* | Lewis, W. H. | K | 6234 | 1962 | Zambia | HiSeq 4000 |
| LG 38 | *Lactuca imbricata* Hiern | Sanane, M. | K | 281 | 1968 | Zambia | HiSeq 4000 |
| LG 39 | *L. imbricata* | Hilliard, O. M. & Burrt, B. L. | E | 4120 | 1967 | Malawi | HiSeq 4000 |
| LG 40 | *Lactuca indica* L. | Zhu, S. X. | ZZU | 2010-1191 | 2010 | China | HiSeq 2000 |
| LG 41 | *L. indica* | Adams, C. D. | BM | 8154 | - | Georgia | HiSeq 4000 |
| LG 42 | *L. indica* | De Wilde, W. J. J. O. | WAG | 2457 | 1905 | Cameroon | HiSeq 2000 |
| LG 43 | *Lactuca inermis* Forssk. | Hull, H. | E | 481 | 1995 | South Africa | HiSeq 4000 |
| LG 44 | *Lactuca intricata* Boiss. | Anonym | E | - | - | - | HiSeq 4000 |
| LG 45 | *L. intricata* | Davis, P. H. | K | 13354 | 1947 | Turkey | HiSeq 4000 |
| LG 46 | *Lactuca lasiorhiza* (O.Hoffm.) C.Jeffrey | Phillips, E. | K | 64 | 1975 | Malawi | HiSeq 4000 |
| LG 47 | *Lactuca longispicata* De Wild. | Richards, H. M. | K | 4694 | 1955 | Zambia | HiSeq 4000 |
| LG 48 | *Lactuca ludoviciana* (Nutt.) Riddell | Wilhelm, S. | BM | 10563 | 1920 | USA | HiSeq 4000 |
| LG 49 | *Lactuca mulgedioides* Boiss. & Kotschy ex Boiss. | Davis | E | 47122 | 1996 | Turkey | HiSeq 4000 |
| LG 50 | *Lactuca mwinilungensis* G.V.Pope | Richards, H. M. | K | 17231 | 1962 | Zambia | HiSeq 4000 |
| LG 51 | *Lactuca oblongifolia* Nutt. | Wojtas, W. A. | E | 667 | 1979 | Canada | HiSeq 4000 |
| LG 52 | *L. oblongifolia* | Cody, W. J. & Myatte, J. M. | E | 8714 | 1955 | Canada | HiSeq 4000 |
| LG 53 | *Lactuca orientalis* Boiss. | Koie, M. | E | 3437 | 1949 | Afghanistan | HiSeq 4000 |
| LG 54 | *Lactuca palmensis* Bolle | Murray, R. P. | BM | - | 1892 | Canary islands | HiSeq 4000 |
| LG 55 | *Lactuca paradoxa* Sch.Bip. ex A.Rich. | Friis, I. | WAG | - | - | - | HiSeq 2000 |
| LG 56 | *Lactuca perennis* L. | Thanpin, A. | E | 13913 | 1977 | France | HiSeq 4000 |
| LG 57 | *L. perennis* | Davis, D. & Sutton, S. | E | D65944 | 1979 | Italy | HiSeq 4000 |
| LG 58 | *Lactuca picridiformis* Boiss. | Rechinger, K.H. | E | 36813 | 1967 | Afghanistan | HiSeq 4000 |
| LG 59 | *Lactuca plumieri* Gren. & Godr. | Jcuroie, H. | E | 64 | 1896 | - | HiSeq 4000 |
| LG 60 | *Lactuca praecox* R.E.Fr. | Mutimishi, J. M. | K | 2675 | 1968 | Zambia | HiSeq 4000 |
| LG 61 | *Lactuca praevia* C.D.Adams | Simons, E. L. A. N. | K | 855 | 2012 | Guinea | HiSeq 4000 |
| LG 62 | *Lactuca quercina*[L.](https://www.ipni.org/n/228200-1) | Dvorakova, M., Smejkal, M. & Vicherek, J. | E | - | 1966 | Czech | HiSeq 4000 |
| LG 63 | *L. quercina* | Callier, A. | BM | 659 | - | USA | HiSeq 4000 |
| LG 64 | *Lactuca racemosa* Willd. | Davis | E | 46524 | 1966 | Turkey | HiSeq 4000 |
| LG 65 | *Lactuca raddeana* Myaxim. | Zhu, S. X. | ZZU | 09-208 | 2009 | China | HiSeq 2000 |
| LG 66 | *Lactuca reviersii* Litard. & Myaire | Anonym | BM | - | - | - | HiSeq 4000 |
| LG 67 | *Lactuca saligna* L. | Sukhorukov, A. | E | 191 | 2010 | Russia | HiSeq 4000 |
| LG 68 | *L. saligna* | Koopman, W. J. M. | WAG | 15705 | 1991 | Georgia | HiSeq 2000 |
| LG 69 | *Lactuca scarioloides* Boiss. | Renz, J. | E | 47626 | 1974 | Iran | HiSeq 4000 |
| LG 70 | *Lactuca schweinfurthii* Oliv. & Hiern | Daramola, B. O. | K | 62749 | 1969 | Nigeria | HiSeq 4000 |
| LG 71 | *Lactuca altaica* Fisch. & C.A.Mey. | Koopman, W. J. M. | WAG | 15711 | 1995 | Georgia | HiSeq 2000 |
| LG 72 | *Lactuca serriola* L. | Fu, S. M. | ZZU | 718 | 2019 | China | Novaseq |
| LG 73 | *Lactuca setosa* Stebbins ex C.Jeffrey | Von Blittersdorff, R. | B | - | 2011 | Tanzania | HiSeq 2000 |
| LG 74 | *Lactuca sibirica* Benth. ex Myaxim. | Pavvo, S. J. | E | - | 1963 | Finland | HiSeq 4000 |
| LG 75 | *L. sibirica* | Myarjatta, I. | E | NJ3 | 1977 | Finland | HiSeq 4000 |
| LG 76 | *Lactuca tatarica* C.A.Mey. | Hamsa, H. E. | E | - | 1969 | Armenia | HiSeq 4000 |
| LG 77 | *L. tatarica* | Hedge, I. C. & Wendelbo, P. | E | 4697 | 1962 | Afghanistan | HiSeq 4000 |
| LG 78 | *Lactuca tenerrima* Pourr. | Gardner, M. F. & Knees, S. G. | E | 169 | 1981 | Spain | HiSeq 4000 |
| LG 79 | *L. tenerrima* | Davis | E | 54935 | 1973 | Morocco | HiSeq 4000 |
| LG 80 | *Lactuca tetrantha* B.L.Burtt & P.H.Davis | Kennedy, E. W. | K | 1483 | 1938 | Cyprus | HiSeq 4000 |
| LG 81 | *Lactuca triangulata* Myaxim. | Kim, H.S. & Jung, S. | PE | TA185 | 2000 | Korea | HiSeq Xten |
| LG 82 | *Lactuca tysonii* (E.Phillips) C.Jeffrey | Rain, M. | E | - | 1969 | - | HiSeq 4000 |
| LG 83 | *Lactuca undulata* Ledeb. | Anonym | PE | 10425 | 1959 | China | HiSeq Xten |
| LG 84 | *Lactuca variabilis* Bornm. | Hiller, A. G. | E | 6186 | 1976 | Turkey | HiSeq 4000 |
| LG 85 | *Lactuca viminea* (L.) J.Presl & C.Presl | Davis, P. H. | E | 40101 | 1964 | Italy | HiSeq 4000 |
| LG 86 | *Lactuca virosa* L. | Anonym | CGN | 09364 | 2013 | Iran | HiSeq 2000 |
| LG 87 | *Melanoseris decipiens* (C.B.Clarke) N.Kilian & Ze H.Wang | Polunin, O. V. | E | 14656 | 1905 | - | HiSeq 4000 |
| LG 88 | *Notoseris macilenta* (Vaniot & H.Lév.) N.Kilian | Lu, Y. | ZZU | 1803 | 2012 | China | Novaseq |
| LG 89 | *Notoseris macilenta* | Lu, Y. & Jin, B. | ZZU | 2012-1823 | 2012 | China | Novaseq |
| LG 90 | *Notoseris triflora* (Hemsl.) C.Shih | Lu, Y. & Jin, B. | ZZU | 2012-1856 | 2012 | China | Novaseq |
| LG 91 | *Paraprenanthes diversifolia* (Vaniot) N.Kilian | Lu, Y. | ZZU | 1801 | 2012 | China | Novaseq |
| LG 92 | *Paraprenanthes diversifolia* | Lu, Y. & Jin, B. | ZZU | 2012-1845 | 2012 | China | Novaseq |
| LG 93 | *Paraprenanthes melanantha* (Franch.) Ze H.Wang | Xiong, J. H. & Zhou, T. L. | PE | 92336 | 1957 | China | HiSeq Xten |
| LG 94 | *Paraprenanthes sororia* (Miq.) C.Shih | Chen, B. | CSH | 08575 | 2015 | China | Novaseq |
| LG 95 | *Paraprenanthes yunnanensis* (Franch.) C.Shih | Zhu, S. X. & Kang, Y. & Gao, T. G. | PE | 76 | 2002 | China | Novaseq |
| LG 96 | *Prenanthes purpurea* L. | Wieringa, J. J. | WAG | 5375 | 1905 | France | HiSeq 2000 |

Note: * *C. auriculiformis* and *C. azurea* have been transferred to *Kovalevskiella* but not formally renamed (Kilian et al. 2017); - indicates missing information.

**Supplementary Table 2.** Information of downloaded plastome sequences.

| Species | Source | Accession No. | Serial No. |
| --- | --- | --- | --- |
| *Cichorium intybus* L. | NCBI | NC043842 | - |
| *Gelasia hirsuta* (Gouan) Zaika, Sukhor. & N.Kilian | DOI:10.1093/sysbio/syy022 | - | - |
| *Lactuca aculeata* Boiss. | CNGBdb | CNA0014206 | TKI 464 |
| *Lactuca altaica* Fisch. & C.A.Mey. | CNGBdb | CNA0014207 | TKI 466 |
| *Lactuca canadensis* L. | CNGBdb | CNA0014209 | TKI 473 |
| *Lactuca georgica* Grossh. | CNGBdb | CNA0014205 | TKI 449 |
| *L. indica* L. | CNGBdb | CNA0014211 | TKI 477 |
| *Lactuca palmensis* Bolle | CNGBdb | CNA0014213 | TKI 494 |
| *Lactuca saligna* L. | CNGBdb | CNA0014203 | TKI 342 |
| *Lactuca sativa* L. | NCBI | AP007232 | - |
| *L. sativa* | NCBI | DQ383816 | - |
| *L. sativa* | NCBI | MT162684 | - |
| *Lactuca serriola* L. | CNGBdb | CNA0014202 | TKI 340 |
| *Lactuca virosa* L. | CNGBdb | CNA0014204 | TKI 404 |
| *Pseudopodospermum hispanicum* (L.) Zaika, Sukhor. & N.Kilian | DOI:10.1093/sysbio/syy022 | - | - |

**Supplementary Table 3.** Information of downloaded ITS sequences.

| Species | Source | Accession No. |
| --- | --- | --- |
| *Cicerbita hispida* Beauverd | NCBI | KF485547 |
| *Cicerbita kovalevskiana* Kirp. | NCBI | LT721945 |
| *Cicerbita roborowskii* Beauverd | NCBI | LT721921 |
| *Cicerbita rosea* Krasch. ex Kovalevsk. | NCBI | LT721989 |
| *Cicerbita zeravschanica* Popov | NCBI | LT721972 |
| *Cichorium intybus* L. | NCBI | AJ746408 |
| *Gelasia hirsuta* (Gouan) Zaika, Sukhor. & N.Kilian | NCBI | KU586735 |
| *Lactuca acanthifolia* Benth. & Hook.f. | NCBI | LT722007 |
| *Lactuca alaica* Kovalevsk. | NCBI | LT722033 |
| *Lactuca alpestris* (Gand.) Rech.f. | NCBI | LT721981 |
| *Lactuca aurea* (Sch.Bip. ex Vis. & Pančić) Stebbins | NCBI | LT722039 |
| *Lactuca attenuata* Stebbins | NCBI | LT721984 |
| *Lactuca calophylla* C.Jeffrey | NCBI | LT721968 |
| *Lactuca deltoidea* DC. ex C.A.Mey. | NCBI | LT721937 |
| *Lactuca fenzlii* N.Kilian & Greuter | NCBI | LT721953 |
| *Lactuca macrophylla* A.Gray | NCBI | LT721959 |
| *Lactuca mira* Pavlov | NCBI | LT721920 |
| *Lactuca sativa* L. | NCBI | AJ633337 |
| *Lactuca schulzeana* Büttner | NCBI | LT721983 |
| *Lactuca tuberosa* A.Chev. | NCBI | LT721940 |
| *Lactuca ugandensis* C.Jeffrey | NCBI | LT721969 |
| *Lactuca watsoniana* Trel. | NCBI | KY067185 |
| *Notoseris henryi* (Dunn) C.Shih | NCBI | KF485591 |
| *Notoseris khasiana* (C.B.Clarke) N.Kilian | NCBI | KF485601 |
| *Notoseris yakoensis* (Jeffrey) N.Kilian | NCBI | KF485589 |
| *Paraprenanthes meridionalis* (C.Shih) Sennikov | NCBI | KF485571 |
| *Paraprenanthes oligolepis* (C.C.Chang ex C.Shih) Ze H.Wang | NCBI | KF485555 |
| *Paraprenanthes prenanthoides* (Hemsl.) C.Shih | NCBI | KF485570 |
| *Paraprenanthes triflora* (C.C.Chang & C.Shih) Ze H.Wang & N.Kilian | NCBI | LT722011 |
| *Lactuca parishii* Craib ex Hosseus | NCBI | KF485563 |
| *Paraprenanthes wilsonii* (C.C.Chang) Ze H.Wang | NCBI | KF485584 |
| *Pseudopodospermum hispanicum* (L.) Zaika, Sukhor. & N.Kilian | NCBI | LT721963 |

**Supplementary Table 4.** Information of ninety-six newly sequenced plastomes of *Lactuca* and its related species.

| Species | Length (bp) | | | | | | | GC content (%) | | | | Gene No. | | | | GenBank  accession |
| --- | --- | --- | --- | --- | --- | --- | --- | --- | --- | --- | --- | --- | --- | --- | --- | --- |
|  | Total | | LSC | SSC | | IR | | Total | LSC | SSC | IR | Total* | CDS | rRNA | tRNA |  |
| *A. triquetra* LG 1 | 152,990 | 84,328 | | | 18,508 | | 25,077 | 37.6 | 35.7 | 31.2 | 43.1 | 132(2) | 87 | 8 | 37 | ON782474 |
| *C. alpina* LG 2 | 149,394 | 84,171 | | | 15,291 | | 24,966 | 37.9 | 35.8 | 31.6 | 43.2 | 129(5) | 84 | 8 | 37 | ON782475 |
| *C. auriculiformis* LG 3 | 152,618 | 83,968 | | | 18,448 | | 25,101 | 37.6 | 35.8 | 31.2 | 43.1 | 131(3) | 86 | 8 | 37 | ON782476 |
| *C. azurea* LG 4 | 152,764 | 84,088 | | | 18,484 | | 25,096 | 37.6 | 35.8 | 31.2 | 43.1 | 131(3) | 86 | 8 | 37 | ON782477 |
| *C. azurea* LG 5 | 152,733 | 84,070 | | | 18,483 | | 25,090 | 37.6 | 35.8 | 31.2 | 43.1 | 132(2) | 87 | 8 | 37 | ON782478 |
| *C. cyprica* LG 6 | 152,742 | 84,114 | | | 18,488 | | 25,070 | 37.7 | 35.8 | 31.3 | 43.1 | 132(2) | 87 | 8 | 37 | ON782479 |
| *C. rechingeriana* LG 7 | 152,701 | 84,074 | | | 18,487 | | 25,070 | 37.7 | 35.8 | 31.2 | 43.1 | 132(2) | 87 | 8 | 37 | ON782537 |
| *F. sinensis* LG 8 | 152,858 | 84,349 | | | 18,473 | | 25,018 | 37.8 | 35.9 | 31.5 | 43.2 | 132(2) | 87 | 8 | 37 | ON782480 |
| *L. aculeata* LG 9 | 152,764 | 84,081 | | | 18,617 | | 25,033 | 37.6 | 35.7 | 31.1 | 43.0 | 131(3) | 86 | 8 | 37 | ON782481 |
| *L. aculeata* LG 10 | 152,744 | 84,082 | | | 18,596 | | 25,033 | 37.6 | 35.7 | 31.0 | 43.0 | 131(3) | 86 | 8 | 37 | ON782539 |
| *L. adenophora* LG 11 | 152,366 | 83799 | | | 18,449 | | 25,059 | 37.6 | 35.8 | 31.0 | 43.1 | 129(5) | 84 | 8 | 37 | ON782482 |
| *L. adenophora* LG 12 | 152,455 | 83,849 | | | 18,510 | | 25,048 | 37.6 | 35.8 | 31.1 | 43.1 | 131(3) | 86 | 8 | 37 | ON782483 |
| *L. alatipes* LG 13 | 152,263 | 83,623 | | | 18,470 | | 25,088 | 37.7 | 35.9 | 31.1 | 43.1 | 130(4) | 85 | 8 | 37 | ON782540 |
| *L. biennis* LG 14 | 152,603 | 83,909 | | | 18,608 | | 25,043 | 37.6 | 35.8 | 31.0 | 43.1 | 131(3) | 86 | 8 | 37 | ON782485 |
| *L. boissieri* LG 15 | 152,381 | 84,074 | | | 18,531 | | 24,888 | 37.6 | 35.8 | 31.2 | 43.1 | 132(2) | 87 | 8 | 37 | ON782486 |
| *L. bourgaei* LG 16 | 152,450 | 83,759 | | | 18,551 | | 25,070 | 37.6 | 35.8 | 31.1 | 43.1 | 132(2) | 87 | 8 | 37 | ON782487 |
| *L. bourgaei* LG 17 | 152,439 | 83,752 | | | 18,551 | | 25,068 | 37.6 | 35.8 | 31.1 | 43.1 | 132(2) | 87 | 8 | 37 | ON782488 |
| *L. brachyrrhyncha* LG 18 | 152,641 | 83,928 | | | 18,627 | | 25,043 | 37.6 | 35.8 | 31.0 | 43.1 | 132(2) | 87 | 8 | 37 | ON782489 |
| *L. canadensis* LG 19 | 152,583 | 83,925 | | | 18,610 | | 25,024 | 37.6 | 35.7 | 31.0 | 43.1 | 132(2) | 87 | 8 | 37 | ON782490 |
| *L. crambifolia* LG 20 | 152,599 | 83,935 | | | 18,484 | | 25,090 | 37.6 | 35.8 | 31.1 | 43.1 | 131(3) | 86 | 8 | 37 | ON782491 |
| *L. dissecta* LG 21 | 152,415 | 84,053 | | | 18,664 | | 24,849 | 37.5 | 35.7 | 31.1 | 43.0 | 132(2) | 87 | 8 | 37 | ON782541 |
| *L. dolichophylla* LG 22 | 152,076 | 83,791 | | | 18,577 | | 24,854 | 37.6 | 35.8 | 31.2 | 43.1 | 132(2) | 87 | 8 | 37 | ON782492 |
| *L. dumicola* LG 23 | 152,911 | 84,271 | | | 18,520 | | 25,060 | 37.6 | 35.7 | 31.0 | 43.1 | 128(6) | 83 | 8 | 37 | ON782542 |
| *L. floridana* LG 24 | 152,324 | 83,647 | | | 18,589 | | 25,044 | 37.6 | 35.8 | 31.0 | 43.1 | 132(2) | 87 | 8 | 37 | ON782493 |
| *L. formosana* LG 25 | 152,773 | 84,056 | | | 18,551 | | 25,083 | 37.6 | 35.7 | 31.0 | 43.1 | 130(4) | 85 | 8 | 37 | ON782494 |
| *L. georgica* LG 26 | 152,731 | 84,189 | | | 18,484 | | 25,029 | 37.5 | 35.7 | 31.2 | 43.0 | 131(3) | 86 | 8 | 37 | ON782543 |
| *L. georgica* LG 27 | 152,731 | 84,189 | | | 18,484 | | 25,029 | 37.5 | 35.7 | 31.2 | 43.0 | 131(3) | 86 | 8 | 37 | ON782495 |
| *L. glandulifera* LG 28 | 152,560 | 83,976 | | | 18,476 | | 25,054 | 37.6 | 35.8 | 31.1 | 43.1 | 131(3) | 86 | 8 | 37 | ON782544 |
| *L. glandulifera* LG 29 | 152,384 | 83,800 | | | 18,476 | | 25,054 | 37.7 | 35.8 | 31.1 | 43.1 | 131(3) | 86 | 8 | 37 | ON782545 |
| *L. glareosa* LG 30 | 152,370 | 84,034 | | | 18,512 | | 24,912 | 37.6 | 35.8 | 31.1 | 43.1 | 130(4) | 85 | 8 | 37 | ON782496 |
| *L. glauciifolia* LG 31 | 152,547 | 83,931 | | | 18,526 | | 25,045 | 37.6 | 35.7 | 31.1 | 43.1 | 131(3) | 86 | 8 | 37 | ON782497 |
| *L. glauciifolia* LG 32 | 152,544 | 83,928 | | | 18,526 | | 25,045 | 37.6 | 35.7 | 31.1 | 43.1 | 131(3) | 86 | 8 | 37 | ON782498 |
| *L. graminifolia* LG 33 | 152,641 | 83,927 | | | 18,628 | | 25,043 | 37.6 | 35.8 | 31.0 | 43.1 | 132(2) | 87 | 8 | 37 | ON782499 |
| *L. graminifolia* LG 34 | 152,616 | 83,925 | | | 18,605 | | 25,043 | 37.6 | 35.7 | 31.0 | 43.1 | 132(2) | 87 | 8 | 37 | ON782500 |
| *L. hirsuta* LG 35 | 152,600 | 83,925 | | | 18,627 | | 25,024 | 37.6 | 35.7 | 31.0 | 43.1 | 132(2) | 87 | 8 | 37 | ON782546 |
| *L. homblei* LG 36 | 152,747 | 84,061 | | | 18,544 | | 25,071 | 37.6 | 35.8 | 31.1 | 43.1 | 128(6) | 83 | 8 | 37 | ON782547 |
| *L. homblei* LG 37 | 152,663 | 84,034 | | | 18,505 | | 25,062 | 37.6 | 35.8 | 31.1 | 43.1 | 128(6) | 83 | 8 | 37 | ON782548 |
| *L. imbricata* LG 38 | 152,637 | 83,989 | | | 18,506 | | 25,071 | 37.6 | 35.8 | 31.1 | 43.1 | 128(6) | 83 | 8 | 37 | ON782549 |
| *L. imbricata* LG 39 | 152,619 | 83,980 | | | 18,497 | | 25,071 | 37.6 | 35.8 | 31.1 | 43.1 | 128(6) | 82 | 8 | 37 | ON782550 |
| *L. indic*a LG 40 | 152,767 | 84,109 | | | 18,498 | | 25,080 | 37.5 | 35.7 | 31.0 | 43.1 | 130(4) | 85 | 8 | 37 | ON782501 |
| *L. indica* LG 41 | 152,700 | 83,999 | | | 18,505 | | 25,098 | 37.6 | 35.7 | 31.0 | 43.1 | 130(4) | 85 | 8 | 37 | ON782551 |
| *L. indica* LG 42 | 152,236 | 83,469 | | | 18,555 | | 25,106 | 37.6 | 35.7 | 31.0 | 43.1 | 128(6) | 83 | 8 | 37 | ON782502 |
| *L. inermis* LG 43 | 152,269 | 83,649 | | | 18,490 | | 25,065 | 37.6 | 35.8 | 31.2 | 43.1 | 131(3) | 86 | 8 | 37 | ON782503 |
| *L. intricata* LG 44 | 152,436 | 83,846 | | | 18,486 | | 25,052 | 37.6 | 35.7 | 31.0 | 43.0 | 130(4) | 85 | 8 | 37 | ON782552 |
| *L. intricata* LG 45 | 152,253 | 83,863 | | | 18,326 | | 25,032 | 37.6 | 35.7 | 31.1 | 43.1 | 130(4) | 85 | 8 | 37 | ON782553 |
| *L. lasiorhiza* LG 46 | 152,746 | 84,109 | | | 18,501 | | 25,068 | 37.6 | 35.8 | 31.1 | 43.1 | 127(7) | 82 | 8 | 37 | ON782504 |
| *L. longispicata* LG 47 | 152,617 | 83,945 | | | 18,524 | | 25,074 | 37.6 | 35.8 | 31.0 | 43.1 | 128(6) | 83 | 8 | 37 | ON782554 |
| *L. ludoviciana* LG 48 | 152,626 | 83,913 | | | 18,627 | | 25,043 | 37.6 | 35.7 | 31.0 | 43.1 | 132(2) | 87 | 8 | 37 | ON782505 |
| *L. mulgedioides* LG 49 | 152,386 | 84,075 | | | 18,537 | | 24,887 | 37.6 | 35.8 | 31.1 | 43.1 | 132(2) | 87 | 8 | 37 | ON782555 |
| *L. mwinilungensis* LG 50 | 152,660 | 84,055 | | | 18,505 | | 25,050 | 37.6 | 35.8 | 31.0 | 43.1 | 128(6) | 83 | 8 | 37 | ON782556 |
| *L. oblongifolia* LG 51 | 152,591 | 83,977 | | | 18,540 | | 25,037 | 37.6 | 35.7 | 31.2 | 43.1 | 131(3) | 86 | 8 | 37 | ON782557 |
| *L. oblongifolia* LG 52 | 152,561 | 83,945 | | | 18,546 | | 25,035 | 37.6 | 35.7 | 31.2 | 43.1 | 132(2) | 87 | 8 | 37 | ON782506 |
| *L. orientalis* LG 53 | 152,289 | 83,960 | | | 18,539 | | 24,895 | 37.5 | 35.6 | 31 | 43.1 | 126(8) | 81 | 8 | 37 | ON782558 |
| *L. palmensis* LG 54 | 152,510 | 83,894 | | | 18,474 | | 25,071 | 37.6 | 35.8 | 31.2 | 43.1 | 132(2) | 87 | 8 | 37 | ON782507 |
| *L. paradoxa* LG 55 | 152,700 | 84,097 | | | 18,493 | | 25,055 | 37.6 | 35.8 | 31.1 | 43.1 | 131(3) | 86 | 8 | 37 | ON782508 |
| *L. perennis* LG 56 | 152,374 | 83,794 | | | 18,520 | | 25,030 | 37.6 | 35.7 | 31.1 | 43.0 | 128(6) | 83 | 8 | 37 | ON782509 |
| *L. perennis* LG 57 | 152,394 | 83,965 | | | 18,441 | | 24,994 | 37.6 | 35.7 | 31.2 | 43.0 | 127(7) | 82 | 8 | 37 | ON782559 |
| *L. picridiformis* LG 58 | 152,679 | 84,035 | | | 18,472 | | 25,086 | 37.6 | 35.8 | 31.1 | 43.0 | 132(2) | 87 | 8 | 37 | ON782510 |
| *L. plumieri* LG 59 | 152,274 | 83,600 | | | 18,582 | | 25,046 | 37.6 | 35.8 | 31.1 | 43.1 | 132(2) | 87 | 8 | 37 | ON782511 |
| *L. praecox* LG 60 | 152,621 | 84,002 | | | 18,499 | | 25,060 | 37.6 | 35.8 | 31.0 | 43.1 | 128(6) | 83 | 8 | 37 | ON782560 |
| *L. praevia* LG 61 | 152,781 | 84,115 | | | 18,526 | | 25,070 | 37.6 | 35.8 | 31.1 | 43.1 | 127(7) | 82 | 8 | 37 | ON782561 |
| *L. quercina* LG 62 | 152,676 | 83,992 | | | 18,560 | | 25,062 | 37.6 | 35.7 | 31.1 | 43.1 | 132(2) | 87 | 8 | 37 | ON782512 |
| *L. quercina* LG 63 | 152,679 | 83,995 | | | 18,560 | | 25,062 | 37.6 | 35.7 | 31.1 | 43.1 | 132(2) | 87 | 8 | 37 | ON782513 |
| *L. racemosa* LG 64 | 152,460 | 83,810 | | | 18,530 | | 25,060 | 37.6 | 35.8 | 31.1 | 43.0 | 129(5) | 84 | 8 | 37 | ON782562 |
| *L. raddeana* LG 65 | 152,724 | 84,032 | | | 18,526 | | 25,083 | 37.6 | 35.7 | 31.0 | 43.1 | 130(4) | 85 | 8 | 37 | ON782514 |
| *L. reviersii* LG 66 | 152,947 | 84,105 | | | 18,780 | | 25,031 | 37.5 | 35.6 | 30.7 | 43.0 | 129(5) | 84 | 8 | 37 | ON782538 |
| *L. saligna* LG 67 | 152,807 | 84,176 | | | 18,561 | | 25,035 | 37.5 | 35.7 | 31.0 | 43.0 | 131(3) | 86 | 8 | 37 | ON782515 |
| *L. saligna* LG 68 | 152,530 | 83,809 | | | 18,647 | | 25,037 | 37.5 | 35.7 | 31.1 | 43.0 | 130(4) | 85 | 8 | 37 | ON782563 |
| *L. sativa* AP007232 | 152,765 | 84,103 | | | 18,596 | | 25,033 | 37.5 | 35.7 | 31.0 | 43.0 | 131(3) | 86 | 8 | 37 | AP007232 |
| *L. sativa* DQ383816 | 152,772 | 84,105 | | | 18,599 | | 25,034 | 37.5 | 35.7 | 31.1 | 43.0 | 130(4) | 85 | 8 | 37 | DQ383816 |
| *L. sativa* MT162684 | 152,744 | 84,103 | | | 18,575 | | 25,033 | 37.5 | 35.7 | 31.0 | 43.0 | 131(3) | 86 | 8 | 37 | MT162684 |
| *L. scarioloides* LG 69 | 152,684 | 84,217 | | | 18,585 | | 24,941 | 37.5 | 35.7 | 31.1 | 43.0 | 130(4) | 85 | 8 | 37 | ON782516 |
| *L. schweinfurthii* LG 70 | 152,610 | 83,958 | | | 18,522 | | 25,065 | 37.6 | 35.8 | 31.1 | 43.1 | 128(6) | 83 | 8 | 37 | ON782564 |
| *L. altaica* LG 71 | 152,760 | 84,095 | | | 18,599 | | 25,033 | 37.5 | 35.7 | 31.0 | 43.0 | 131(3) | 86 | 8 | 37 | ON782484 |
| *L. serriola* LG 72 | 152,730 | 84,066 | | | 18,598 | | 25,033 | 37.6 | 35.7 | 31.1 | 43.0 | 131(3) | 86 | 8 | 37 | ON782517 |
| *L. setosa* LG 73 | 152,754 | 84,028 | | | 18,522 | | 25,102 | 37.6 | 35.8 | 31.1 | 43.1 | 127(7) | 82 | 8 | 37 | ON782518 |
| *L. sibirica* LG 74 | 152,586 | 83,998 | | | 18,532 | | 25,028 | 37.6 | 35.7 | 31.1 | 43.1 | 132(2) | 87 | 8 | 37 | ON782519 |
| *L. sibirica* LG 75 | 152,563 | 83,975 | | | 18,532 | | 25,028 | 37.6 | 35.7 | 31.2 | 43.1 | 132(2) | 87 | 8 | 37 | ON782520 |
| *L. tatarica* LG 76 | 152,380 | 83,823 | | | 18,549 | | 25,004 | 37.6 | 35.7 | 31.1 | 43.1 | 132(2) | 87 | 8 | 37 | ON782521 |
| *L. tatarica* LG 77 | 152,371 | 83,826 | | | 18,535 | | 25,005 | 37.6 | 35.7 | 31.2 | 43.1 | 132(2) | 87 | 8 | 37 | ON782522 |
| *L. tenerrima* LG 78 | 152,621 | 83,939 | | | 18,538 | | 25,072 | 37.6 | 35.8 | 31.2 | 43.1 | 132(2) | 87 | 8 | 37 | ON782523 |
| *L. tenerrima* LG 79 | 152,623 | 83,934 | | | 18,543 | | 25,073 | 37.6 | 35.8 | 31.2 | 43.1 | 132(2) | 87 | 8 | 37 | ON782524 |
| *L. tetrantha* LG 80 | 152,656 | 84,013 | | | 18,691 | | 24,976 | 37.5 | 35.6 | 30.8 | 43.1 | 130(4) | 85 | 8 | 37 | ON782525 |
| *L. triangulata* LG 81 | 152,633 | 83,959 | | | 18,504 | | 25,085 | 37.6 | 35.7 | 31.0 | 43.1 | 130(4) | 85 | 8 | 37 | ON782526 |
| *L. tysonii* LG 82 | 152,361 | 83,962 | | | 18,587 | | 24,906 | 37.7 | 35.9 | 31.3 | 43.2 | 129(5) | 84 | 8 | 37 | ON782565 |
| *L. undulata* LG 83 | 152,340 | 83,767 | | | 18,479 | | 25,047 | 37.6 | 35.7 | 31.1 | 43.0 | 131(3) | 86 | 8 | 37 | ON782527 |
| *L. variabilis* LG 84 | 152,355 | 84,046 | | | 18,545 | | 24,882 | 37.6 | 35.8 | 31.1 | 43.1 | 132(2) | 87 | 8 | 37 | ON782566 |
| *L. viminea* LG 85 | 152,823 | 84,050 | | | 18,709 | | 25,032 | 37.5 | 35.6 | 30.8 | 43.0 | 129(5) | 84 | 8 | 37 | ON782567 |
| *L. virosa* LG 86 | 152,497 | 83,953 | | | 18,484 | | 25,030 | 37.5 | 35.7 | 31.1 | 43.0 | 131(3) | 86 | 8 | 37 | ON782528 |
| *M. decipiens* LG 87 | 152,474 | 83,863 | | | 18,627 | | 24,992 | 37.7 | 35.9 | 31.2 | 43.1 | 132(2) | 87 | 8 | 37 | ON782568 |
| *N. macilenta* LG 88 | 152,174 | 83,611 | | | 18,509 | | 25,027 | 37.7 | 35.9 | 31.1 | 43.1 | 132(2) | 87 | 8 | 37 | ON782529 |
| *N. macilenta* LG 89 | 152,175 | 83,612 | | | 18,509 | | 25,027 | 37.7 | 35.9 | 31.1 | 43.1 | 132(2) | 87 | 8 | 37 | ON782530 |
| *N. triflora* LG 90 | 152,225 | 83,668 | | | 18,515 | | 25,021 | 37.7 | 35.9 | 31.1 | 43.1 | 132(2) | 87 | 8 | 37 | ON782531 |
| *P. diversifolia* LG 91 | 152,288 | 83,714 | | | 18,496 | | 25,039 | 37.7 | 35.9 | 31.1 | 43.1 | 133(1) | 86 | 8 | 37 | ON782532 |
| *P. diversifolia* LG 92 | 152,285 | 83,707 | | | 18,498 | | 25,040 | 37.7 | 35.9 | 31.1 | 43.1 | 132(2) | 87 | 8 | 37 | ON782533 |
| *P. melanantha* LG 93 | 152,183 | 83,674 | | | 18,343 | | 25,083 | 37.7 | 35.9 | 31.2 | 43.1 | 132(2) | 87 | 8 | 37 | ON782534 |
| *P. sororia* LG 94 | 152,307 | 83,717 | | | 18,500 | | 25,045 | 37.7 | 35.9 | 31.1 | 43.1 | 132(2) | 87 | 8 | 37 | ON782535 |
| *P. yunnanensis* LG 95 | 152,200 | 83,671 | | | 18,451 | | 25,039 | 37.7 | 35.9 | 31.1 | 43.1 | 132(2) | 87 | 8 | 37 | ON782536 |
| *Prenanthes purpurea* LG 96 | 152,820 | 84,091 | | | 18,505 | | 25,112 | 37.7 | 35.9 | 31.3 | 43.1 | 132(2) | 87 | 8 | 37 | ON782569 |

Note: * means the number in parentheses is pseudogene number.

**Supplementary Table 5.** Pseudogene information of *Lactuca* and related species.

| Species | Insertion or deletion | Premature termination codon | Overdue termination codon | Incomplete copy near boundaries |
| --- | --- | --- | --- | --- |
| *A. triquetra* LG 1 |  |  |  | *rps19*, *ycf1* |
| *C. alpina* LG 2 | *rps19*, *ycf1*, *ndhF* |  |  | *rps19*, *ycf1* |
| *C. auriculiformis* LG 3 | *rps15* |  |  | *rps19*, *ycf1* |
| *C. azurea* LG 4 | *matK* |  |  | *rps19*, *ycf1* |
| *C. azurea* LG 5 |  |  |  | *rps19*, *ycf1* |
| *C. cyprica* LG 6 |  |  |  | *rps19*, *ycf1* |
| *C. rechingeriana* LG 7 |  |  |  | *rps19*, *ycf1* |
| *F. sinensis* LG 8 |  |  |  | *rps19*, *ycf1* |
| *L. aculeata* LG 10 | *ndhF* |  |  | *rps19*, *ycf1* |
| *L. aculeata* LG 9 | *ndhF* |  |  | *rps19*, *ycf1* |
| *L. aculeata* TKI 464 | *ndhF* |  |  | *rps19*, *ycf1* |
| *L. adenophora* LG 11 | *rpl22*, *rpoC2* |  | *rps16* | *rps19*, *ycf1* |
| *L. adenophora* LG 12 | *rpl22* |  |  | *rps19*, *ycf1* |
| *L. alatipes* LG 13 | *infA* |  | *ndhF* | *rps19*, *ycf1* |
| *L. altaica* TKI 466 | *ndhF* |  |  | *rps19*, *ycf1* |
| *L. biennis* LG 14 |  | *ndhF* |  | *rps19*, *ycf1* |
| *L. boissieri* LG 15 |  |  |  | *rps19*, *ycf1* |
| *L. bourgaei* LG 16 |  |  |  | *rps19*, *ycf1* |
| *L. bourgaei* LG 17 |  |  |  | *rps19*, *ycf1* |
| *L. brachyrrhyncha* LG 18 |  |  |  | *rps19*, *ycf1* |
| *L. canadensis* LG 19 |  |  |  | *rps19*, *ycf1* |
| *L. canadensis* TKI 473 |  |  |  | *rps19*, *ycf1* |
| *L. crambifolia* LG 20 | *rps16* |  |  | *rps19*, *ycf1* |
| *L. dissecta* LG 21 |  |  |  | *rps19*, *ycf1* |
| *L. dolichophylla* LG 22 |  |  |  | *rps19*, *ycf1* |
| *L. dumicola* LG 23 | *matK* | *psaJ*, *ycf15* |  | *rps19*, *ycf1* |
| *L. floridana* LG 24 |  |  |  | *rps19*, *ycf1* |
| *L. formosana* LG 25 | *ycf2* |  |  | *rps19*, *ycf1* |
| *L. georgica* LG 26 | *ndhF* |  |  | *rps19*, *ycf1* |
| *L. georgica* LG 27 | *ndhF* |  |  | *rps19*, *ycf1* |
| *L. georgica* TKI 449 | *ndhF* |  |  | *rps19*, *ycf1* |
| *L. glandulifera* LG 28 | *rpoC2* |  |  | *rps19*, *ycf1* |
| *L. glandulifera* LG 29 | *rpoC2* |  |  | *rps19*, *ycf1* |
| *L. glareosa* LG 30 | *ycf2* |  |  | *rps19*, *ycf1* |
| *L. glauciifolia* LG 31 | *clpP* |  |  | *rps19*, *ycf1* |
| *L. glauciifolia* LG 32 | *clpP* |  |  | *rps19*, *ycf1* |
| *L. graminifolia* LG 33 |  |  |  | *rps19*, *ycf1* |
| *L. graminifolia* LG 34 |  |  |  | *rps19*, *ycf1* |
| *L. hirsuta* LG 35 |  |  |  | *rps19*, *ycf1* |
| *L. homblei* LG 36 | *matK* | *psaJ*, *ycf15* |  | *rps19*, *ycf1* |
| *L. homblei* LG 37 | *matK* | *psaJ*, *ycf15* |  | *rps19*, *ycf1* |
| *L. imbricata* LG 38 | *matK* | *psaJ*, *ycf15* |  | *rps19*, *ycf1* |
| *L. imbricata* LG 39 | *rps15*, *matK* | *psaJ*, *ycf15* |  | *rps19*, *ycf1* |
| *L. indica* LG 40 | *rpoB*, *ycf2* |  |  | *rps19*, *ycf1* |
| *L. indica* LG 41 | *ycf2* |  |  | *rps19*, *ycf1* |
| *L. indica* LG 42 | *rps19*, *ycf2* | *ycf1* |  | *rps19*, *ycf1* |
| *L. indica* TKI 477 | *ycf2* |  |  | *rps19*, *ycf1* |
| *L. inermis* LG 43 | *rps15* |  |  | *rps19*, *ycf1* |
| *L. intricata* LG 44 | *clpP*, *rpl33* |  |  | *rps19*, *ycf1* |
| *L. intricata* LG 45 | *clpP* |  | *ndhF* | *rps19*, *ycf1* |
| *L. lasiorhiza* LG 46 | *matK*, *rpoC1* | *psaJ*, *ycf15* |  | *rps19*, *ycf1* |
| *L. longispicata* LG 47 | *matK* | *psaJ*, *ycf15* |  | *rps19*, *ycf1* |
| *L. ludoviciana* LG 48 |  |  |  | *rps19*, *ycf1* |
| *L. mulgedioides* LG 49 |  |  |  | *rps19*, *ycf1* |
| *L. mwinilungensis* LG 50 | *matK* | *psaJ*, *ycf15* |  | *rps19*, *ycf1* |
| *L. oblongifolia* LG 51 | *rps19* |  |  | *rps19*, *ycf1* |
| *L. oblongifolia* LG 52 |  |  |  | *rps19*, *ycf1* |
| *L. orientalis* LG 53 | *ycf2*, *ndhF*, *rpoA* | *ccsA*, *psaJ* |  | *rps19*, *ycf1* |
| *L. palmensis* LG 54 |  |  |  | *rps19*, *ycf1* |
| *L. palmensis* TKI 494 |  |  |  | *rps19*, *ycf1* |
| *L. paradoxa* LG 55 | *rps19* |  |  | *rps19*, *ycf1* |
| *L. perennis* LG 56 | *clpP*, *ndhF*, *rpoC1* |  | *rps3* | *rps19*, *ycf1* |
| *L. perennis* LG 57 | *clpP*, *ndhD*, *ndhF, rpoC1* |  | *rps3* | *rps19*, *ycf1* |
| *L. picridiformis* LG 58 |  |  |  | *rps19*, *ycf1* |
| *L. plumieri* LG 59 |  |  |  | *rps19*, *ycf1* |
| *L. praecox* LG 60 | *matK* | *psaJ*, *ycf15* |  | *rps19*, *ycf1* |
| *L. praevia* LG 61 | *matK*, *ndhD* | *psaJ*, *ycf15* |  | *rps19*, *ycf1* |
| *L. quercina* LG 62 |  |  |  | *rps19*, *ycf1* |
| *L. quercina* LG 63 |  |  |  | *rps19*, *ycf1* |
| *L. racemosa* LG 64 | *rpl22*, *ycf2* |  |  | *rps19*, *ycf1* |
| *L. raddeana* LG 65 | *ycf2* |  |  | *rps19*, *ycf1* |
| *L. reviersii* LG 66 | *ndhF* | *ccsA*, *psaJ* |  | *rps19*, *ycf1* |
| *L. saligna* LG 67 | *ndhF* |  |  | *rps19*, *ycf1* |
| *L. saligna* LG 68 | *ndhF* | *ycf1* |  | *rps19*, *ycf1* |
| *L. saligna* TKI 342 | *ndhF* |  |  | *rps19*, *ycf1* |
| *L. sativa* AP007232 | *ndhF* |  |  | *rps19*, *ycf1* |
| *L. sativa* DQ383816 | *ndhF* | *ycf1* |  | *rps19*, *ycf1* |
| *L. sativa* MT162684 | *ndhF* |  |  | *rps19*, *ycf1* |
| *L. scarioloides* LG 69 | *ndhF*, *ndhK* |  |  | *rps19*, *ycf1* |
| *L. schweinfurthii* LG 70 | *matK*, *rps19* | *psaJ*, *ycf15* |  | *rps19*, *ycf1* |
| *L. altaica* LG 71 | *ndhF* |  |  | *rps19*, *ycf1* |
| *L. serriola* LG 72 | *ndhF* |  |  | *rps19*, *ycf1* |
| *L. serriola* TKI 340 | *ndhF* |  |  | *rps19*, *ycf1* |
| *L. setosa* LG 73 | *matK*, *rps19* | *psaJ*, *ycf15* |  | *rps19*, *ycf1* |
| *L. sibirica* LG 74 |  |  |  | *rps19*, *ycf1* |
| *L. sibirica* LG 75 |  |  |  | *rps19*, *ycf1* |
| *L. tatarica* LG 76 |  |  |  | *rps19*, *ycf1* |
| *L. tatarica* LG 77 |  |  |  | *rps19*, *ycf1* |
| *L. tenerrima* LG 78 |  |  |  | *rps19*, *ycf1* |
| *L. tenerrima* LG 79 |  |  |  | *rps19*, *ycf1* |
| *L. tetrantha* LG 80 | *ndhF* | *ccsA*, *psaJ* |  | *rps19*, *ycf1* |
| *L. triangulata* LG 81 | *ycf2* |  |  | *rps19*, *ycf1* |
| *L. tysonii* LG 82 | *rpoC2* | *psaJ*, *ccsA* |  | *rps19*, *ycf1* |
| *L. undulata* LG 83 | *clpP* |  |  | *rps19*, *ycf1* |
| *L. variabilis* LG 84 |  |  |  | *rps19*, *ycf1* |
| *L. viminea* LG 85 | *ndhF* | *ccsA*, *psaJ* |  | *rps19*, *ycf1* |
| *L. virosa* LG 86 | *ndhF* |  |  | *rps19*, *ycf1* |
| *L. virosa* TKI 404 | *ndhF* |  |  | *rps19*, *ycf1* |
| *M. decipiens* LG 87 |  |  |  | *rps19*, *ycf1* |
| *N. macilenta* LG 88 |  |  |  | *rps19*, *ycf1* |
| *N. macilenta* LG 89 |  |  |  | *rps19*, *ycf1* |
| *N. triflora* LG 90 |  |  |  | *rps19*, *ycf1* |
| *P. diversifolia* LG 91 |  | *ycf1* |  | *rps19*, *ycf1* |
| *P. diversifolia* LG 92 |  |  |  | *rps19*, *ycf1* |
| *P. melanantha* LG 93 |  |  |  | *rps19*, *ycf1* |
| *P. sororia* LG 94 |  |  |  | *rps19*, *ycf1* |
| *P. yunnanensis* LG 95 |  |  |  | *rps19*, *ycf1* |
| *P. purpurea* LG 96 |  |  |  | *rps19*, *ycf1* |

**Supplementary Table 6.** Information of downloaded chloroplast DNA fragments.

| Species | Database | Accession No. | Serial No. |
| --- | --- | --- | --- |
| *Notoseris henryi* (Dunn) C.Shih | NCBI | KF486102; KF486230; KF485974 | LAC 056 |
| *Notoseris khasiana* (C.B.Clarke) N.Kilian | NCBI | KF486112; KF486240; KF485984 | LAC 066 |
| *Notoseris scandens* (Hook.f. ex Benth. & Hook.f.) N.Kilian | NCBI | KF486098; KF486226; KF485970 | LAC 052 |
| *Notoseris yakoensis* (Jeffrey) N.Kilian | NCBI | KF486100; KF486228; KF485972 | LAC 054 |
| *Paraprenanthes oligolepis* (C.C.Chang ex C.Shih) Ze H.Wang | NCBI | KF486066; KF486194; KF485938 | LAC 020 |
| *Paraprenanthes prenanthoides* (Hemsl.) C.Shih | NCBI | KF486081; KF486209; KF485953 | LAC 035 |
| *Paraprenanthes triflora* (C.C.Chang & C.Shih) Ze H.Wang & N.Kilian | NCBI | KF486072; KF486200  KF485944 | LAC 026 |
| *Lactuca parishii* Craib ex Hosseus | NCBI | KF486074; KF486202; KF485946 | LAC 028 |
| *Paraprenanthes wilsonii* (C.C.Chang) Ze H.Wang | NCBI | KF486095; KF486223; KF485967 | LAC 049 |
| *Pseudopodospermum hispanicum* (L.) Zaika, Sukhor. & N.Kilian | NCBI | LT722190; LT722305; LT722420 | LAC 296 |

## Supplementary Figures

**Supplementary Figure 1.** Genetic map of *Lactuca* plastomes. The translation of genes outside the outer circle occurs in a counter-clockwise direction, while the translation of genes inside occurs in a clockwise direction; the dark and light gray colors in the inner circle indicate GC and AT content, respectively; different colors represent different functional categories of genes.

**Supplementary Figure 2.** The alignment of plastomes of 46 *Lactuca* and 13 AE *Lactuca* species. The numbers at the bottom represent the position in the plastomes, while the percentages on the right represent similarity.

**Supplementary Figure 3.** Boundaries of the LSC, SSC, and IRs in plastomes of *Lactuca* and AE *Lactuca* species. JLB: Junction between the LSC and IRb; JSB: Junction between the SSC and IRb; JSA: Junction between the SSC and IRa; JLA: Junction between the LSC and IRa.

**Supplementary Figure 4.** ML phylogenetic tree based on chloroplast DNA fragments. **(A)** Phylogenetic trees with branch length. **(B)** ML phylogenetic tree of *Lactuca* and related genera based on chloroplast DNA fragments; phylogenetic tree of chloroplast DNA fragments was constructed based on *rpl32-trnL-UAG*、*trnL-trnF* and *trnQ-UUG-rps16* combined dataset (Supplementary Table4); the branches of the red pentacle and dotted lines are *L. alatipes* and *L. parishii*; no support values are shown for nodes with full support (BS = 100, PP = 1).

**Supplementary Figure 5.** Phylogenetic tree based on the ITS sequences. **(A)** Phylogenetic tree with branch length. **(B)** ML tree with collapsed clades of *Lactuca* and AE *Lactuca* species, the width of the triangle represents the number of species included; no support values are shown for nodes with full support (BS = 100, PP = 1); clades with low support (BS < 50) were collapsed.

**Supplementary Figure 6.** Phylogenetic tree of ITS sequences of *Lactuca* and AE *Lactuca* species. **(A)** Phylogenetic tree with branch length. **(B)** ML tree, the colors are consistent with **Supplementary Figure 5**, no support values are shown for nodes with full support (BS = 100, PP = 1); clades with low support (BS < 50) were collapsed; the vertical lines on the right represent the number of chromosomes.

**Supplementary Figure 7.** Non-synonymous/synonymous of 61 CDS from the plastoms of 45 *Lactuca* species (excluding *L. alatipes*).

**Supplementary Figure 8** Non-synonymous/synonymous of 73 shared CDS from the plastoms of 12 lettuce gene pool species.

**Supplementary Figure 9.** Ancestral stat biogeographic distribution of Lactucinae taxa, based on BEAST consensus tree topology using plastome data; values at nodes show stat posterior probabilities; information of node number in circles are shown in supplementary file: A: N + Central + E Europe, B: SW +SE Europe, C: N Africa + Macaronesia, D: Tropical Africa, E: S Africa, F: Asia-Temperate W, G: Siberia + Russian Far East, H: Asia-Temperate E, I: Indian Subcontinent, J: Asia-Tropical E, K: N America, L: S America.

# Supplementary Data

Ancestral area estimation of *Lactuca* and related genera.

NODE112:

EVENT MATRIX:

Dispersal:0

Vicariance:0

Extinction:0

Event Route:

K->K^K->K|K

PROBABILITY:

1.0000

NODE113:

EVENT MATRIX:

Dispersal:6

Vicariance:0

Extinction:0

Event Route:

F->F^A^F^G^H->AFGH^A^F^G^H->AFGH|AFGH

PROBABILITY:

1.0000

NODE114:

EVENT MATRIX:

Dispersal:0

Vicariance:1

Extinction:0

Event Route:

FK->F|K

PROBABILITY:

1.0000

NODE115:

EVENT MATRIX:

Dispersal:10

Vicariance:0

Extinction:0

Event Route:

F->F^A^B^F^H^G^I->ABFGHI^A^B^F^H^G^I->ABFHGI|ABFHGI

PROBABILITY:

1.0000

NODE116:

EVENT MATRIX:

Dispersal:1

Vicariance:0

Extinction:0

Event Route:

F->F^F->FK^F->F|FK

PROBABILITY:

1.0000

NODE117:

EVENT MATRIX:

Dispersal:6

Vicariance:0

Extinction:0

Event Route:

F->F^A^B^F^H->ABFH^A^B^F^H->ABFH|ABFH

PROBABILITY:

1.0000

NODE118:

EVENT MATRIX:

Dispersal:0

Vicariance:0

Extinction:0

Event Route:

F->F^F->F|F

PROBABILITY:

1.0000

NODE119:

EVENT MATRIX:

Dispersal:0

Vicariance:0

Extinction:0

Event Route:

F->F^F->F|F

PROBABILITY:

1.0000

NODE120:

EVENT MATRIX:

Dispersal:0

Vicariance:0

Extinction:0

Event Route:

F->F^F->F|F

PROBABILITY:

1.0000

NODE121:

EVENT MATRIX:

Dispersal:8

Vicariance:0

Extinction:0

Event Route:

H->H^D^G^H^I^J->DGHIJ^D^G^H^I^J->DGHIJ|DGHIJ

PROBABILITY:

1.0000

NODE122:

EVENT MATRIX:

Dispersal:4

Vicariance:0

Extinction:0

Event Route:

H->H^H->DGHIJ^H->DGHIJ|H

PROBABILITY:

1.0000

NODE123:

EVENT MATRIX:

Dispersal:4

Vicariance:0

Extinction:0

Event Route:

H->H^H->DGHIJ^H->DGHIJ|H

PROBABILITY:

1.0000

NODE124:

EVENT MATRIX:

Dispersal:2

Vicariance:0

Extinction:0

Event Route:

H->H^H->GHJ^H->H|GHJ

PROBABILITY:

1.0000

NODE125:

EVENT MATRIX:

Dispersal:1

Vicariance:0

Extinction:0

Event Route:

H->H^H->GH^H->HG|H

PROBABILITY:

1.0000

NODE126:

EVENT MATRIX:

Dispersal:0

Vicariance:0

Extinction:0

Event Route:

H->H^H->H|H

PROBABILITY:

1.0000

NODE127:

EVENT MATRIX:

Dispersal:0

Vicariance:1

Extinction:0

Event Route:

FH->H|F

PROBABILITY:

1.0000

NODE128:

EVENT MATRIX:

Dispersal:0

Vicariance:0

Extinction:0

Event Route:

F->F^F->F|F

PROBABILITY:

1.0000

NODE129:

EVENT MATRIX:

Dispersal:0

Vicariance:0

Extinction:0

Event Route:

F->F^F->F|F

PROBABILITY:

1.0000

NODE130:

EVENT MATRIX:

Dispersal:1

Vicariance:0

Extinction:0

Event Route:

F->F^F->FH^F->F|FH

PROBABILITY:

1.0000

NODE131:

EVENT MATRIX:

Dispersal:0

Vicariance:0

Extinction:0

Event Route:

F->F^F->F|F

PROBABILITY:

1.0000

NODE132:

EVENT MATRIX:

Dispersal:0

Vicariance:0

Extinction:0

Event Route:

F->F^F->F|F

PROBABILITY:

1.0000

NODE133:

EVENT MATRIX:

Dispersal:0

Vicariance:0

Extinction:0

Event Route:

F->F^F->F|F

PROBABILITY:

1.0000

NODE134:

EVENT MATRIX:

Dispersal:5

Vicariance:0

Extinction:0

Event Route:

F->F^F^H^I->FHIJ^F^H^I->FHI|FHIJ

PROBABILITY:

1.0000

NODE135:

EVENT MATRIX:

Dispersal:0

Vicariance:0

Extinction:0

Event Route:

F->F^F->F|F

PROBABILITY:

1.0000

NODE136:

EVENT MATRIX:

Dispersal:0

Vicariance:0

Extinction:0

Event Route:

F->F^F->F|F

PROBABILITY:

1.0000

NODE137:

EVENT MATRIX:

Dispersal:0

Vicariance:0

Extinction:0

Event Route:

F->F^F->F|F

PROBABILITY:

1.0000

NODE138:

EVENT MATRIX:

Dispersal:0

Vicariance:0

Extinction:0

Event Route:

F->F^F->F|F

PROBABILITY:

1.0000

NODE139:

EVENT MATRIX:

Dispersal:0

Vicariance:0

Extinction:0

Event Route:

F->F^F->F|F

PROBABILITY:

1.0000

NODE140:

EVENT MATRIX:

Dispersal:0

Vicariance:0

Extinction:0

Event Route:

F->F^F->F|F

PROBABILITY:

1.0000

NODE141:

EVENT MATRIX:

Dispersal:0

Vicariance:0

Extinction:0

Event Route:

F->F^F->F|F

PROBABILITY:

1.0000

NODE142:

EVENT MATRIX:

Dispersal:6

Vicariance:0

Extinction:0

Event Route:

F->F^F->ABCDFHI^F->ABCDFHI|F

PROBABILITY:

1.0000

NODE143:

EVENT MATRIX:

Dispersal:12

Vicariance:0

Extinction:0

Event Route:

F->F^A^B^C^D^F^H^I->ABCDFHI^A^B^C^D^F^H^I->ABCDFHI|ABCDFHI

PROBABILITY:

1.0000

NODE144:

EVENT MATRIX:

Dispersal:6

Vicariance:0

Extinction:0

Event Route:

F->F^F->ABCDFHI^F->ABCDFHI|F

PROBABILITY:

1.0000

NODE145:

EVENT MATRIX:

Dispersal:0

Vicariance:0

Extinction:0

Event Route:

F->F^F->F|F

PROBABILITY:

1.0000

NODE146:

EVENT MATRIX:

Dispersal:0

Vicariance:0

Extinction:0

Event Route:

F->F^F->F|F

PROBABILITY:

1.0000

NODE147:

EVENT MATRIX:

Dispersal:0

Vicariance:1

Extinction:0

Event Route:

BCF->BC|F

PROBABILITY:

0.3333

NODE148:

EVENT MATRIX:

Dispersal:2

Vicariance:0

Extinction:0

Event Route:

F->F^F->BCF^F->F|BCF

PROBABILITY:

0.3333

NODE149:

EVENT MATRIX:

Dispersal:0

Vicariance:0

Extinction:0

Event Route:

F->F^F->F|F

PROBABILITY:

1.0000

NODE150:

EVENT MATRIX:

Dispersal:0

Vicariance:1

Extinction:0

Event Route:

BCF->BC|F

PROBABILITY:

0.3333

NODE151:

EVENT MATRIX:

Dispersal:2

Vicariance:0

Extinction:0

Event Route:

F->F^F->BCF^F->BCF|F

PROBABILITY:

0.3333

NODE152:

EVENT MATRIX:

Dispersal:6

Vicariance:0

Extinction:0

Event Route:

F->F^A^B^C^F->ABCF^A^B^C^F->ABCF|ABCF

PROBABILITY:

1.0000

NODE153:

EVENT MATRIX:

Dispersal:3

Vicariance:0

Extinction:0

Event Route:

F->F^F->ABCF^F->ABCF|F

PROBABILITY:

1.0000

NODE154:

EVENT MATRIX:

Dispersal:0

Vicariance:0

Extinction:0

Event Route:

F->F^F->F|F

PROBABILITY:

1.0000

NODE155:

EVENT MATRIX:

Dispersal:3

Vicariance:0

Extinction:0

Event Route:

BCFH->BCFH^C->ABCFHI^C->C|ABCFIH

PROBABILITY:

0.0833

NODE156:

EVENT MATRIX:

Dispersal:3

Vicariance:0

Extinction:0

Event Route:

CF->CF^F->BCFH^F->F|BCFH

PROBABILITY:

0.0417

NODE157:

EVENT MATRIX:

Dispersal:4

Vicariance:0

Extinction:0

Event Route:

F->F^C^F->CFHI^C^F->CFHI|CF

PROBABILITY:

0.5000

NODE158:

EVENT MATRIX:

Dispersal:0

Vicariance:0

Extinction:0

Event Route:

F->F^F->F|F

PROBABILITY:

1.0000

NODE159:

EVENT MATRIX:

Dispersal:0

Vicariance:0

Extinction:0

Event Route:

F->F^F->F|F

PROBABILITY:

1.0000

NODE160:

EVENT MATRIX:

Dispersal:2

Vicariance:0

Extinction:0

Event Route:

F->F^F^I->FI^F^I->FI|FI

PROBABILITY:

1.0000

NODE161:

EVENT MATRIX:

Dispersal:2

Vicariance:0

Extinction:0

Event Route:

F->F^F->FHI^F->FHI|F

PROBABILITY:

1.0000

NODE162:

EVENT MATRIX:

Dispersal:2

Vicariance:0

Extinction:0

Event Route:

A->A^A^B->AB^A^B->AB|AB

PROBABILITY:

0.5000

NODE163:

EVENT MATRIX:

Dispersal:1

Vicariance:1

Extinction:1

Event Route:

BF->F->AF->A|F

PROBABILITY:

0.2500

NODE164:

EVENT MATRIX:

Dispersal:2

Vicariance:0

Extinction:0

Event Route:

F->F^B^F->BF^B^F->BF|BF

PROBABILITY:

1.0000

NODE165:

EVENT MATRIX:

Dispersal:1

Vicariance:0

Extinction:0

Event Route:

F->F^F->BF^F->F|BF

PROBABILITY:

0.5000

NODE166:

EVENT MATRIX:

Dispersal:0

Vicariance:0

Extinction:0

Event Route:

F->F^F->F|F

PROBABILITY:

1.0000

NODE167:

EVENT MATRIX:

Dispersal:1

Vicariance:0

Extinction:0

Event Route:

K->K^K->KL^K->KL|K

PROBABILITY:

1.0000

NODE168:

EVENT MATRIX:

Dispersal:1

Vicariance:0

Extinction:0

Event Route:

K->K^K->KL^K->KL|K

PROBABILITY:

1.0000

NODE169:

EVENT MATRIX:

Dispersal:1

Vicariance:0

Extinction:0

Event Route:

K->K^K->KL^K->KL|K

PROBABILITY:

1.0000

NODE170:

EVENT MATRIX:

Dispersal:2

Vicariance:0

Extinction:0

Event Route:

K->K^K^L->KL^K^L->KL|KL

PROBABILITY:

1.0000

NODE171:

EVENT MATRIX:

Dispersal:0

Vicariance:0

Extinction:0

Event Route:

K->K^K->K|K

PROBABILITY:

1.0000

NODE172:

EVENT MATRIX:

Dispersal:1

Vicariance:0

Extinction:0

Event Route:

K->K^K->KL^K->KL|K

PROBABILITY:

1.0000

NODE173:

EVENT MATRIX:

Dispersal:0

Vicariance:0

Extinction:0

Event Route:

K->K^K->K|K

PROBABILITY:

1.0000

NODE174:

EVENT MATRIX:

Dispersal:0

Vicariance:0

Extinction:0

Event Route:

K->K^K->K|K

PROBABILITY:

1.0000

NODE175:

EVENT MATRIX:

Dispersal:1

Vicariance:1

Extinction:0

Event Route:

AK->ABK->AB|K

PROBABILITY:

0.3333

NODE176:

EVENT MATRIX:

Dispersal:2

Vicariance:0

Extinction:0

Event Route:

C->C^B^C->BC^B^C->BC|BC

PROBABILITY:

1.0000

NODE177:

EVENT MATRIX:

Dispersal:0

Vicariance:0

Extinction:0

Event Route:

C->C^C->C|C

PROBABILITY:

1.0000

NODE178:

EVENT MATRIX:

Dispersal:0

Vicariance:0

Extinction:0

Event Route:

C->C^C->C|C

PROBABILITY:

1.0000

NODE179:

EVENT MATRIX:

Dispersal:2

Vicariance:0

Extinction:0

Event Route:

CDF->CDF^C->CDEF^C->CDEF|C

PROBABILITY:

0.2000

NODE180:

EVENT MATRIX:

Dispersal:2

Vicariance:0

Extinction:0

Event Route:

CFI->CFI^F->CDFI^F->FI|CDF

PROBABILITY:

0.0667

NODE181:

EVENT MATRIX:

Dispersal:3

Vicariance:1

Extinction:1

Event Route:

BFK->FK->ACFIK->CFI|AK

PROBABILITY:

0.0051

NODE182:

EVENT MATRIX:

Dispersal:2

Vicariance:0

Extinction:0

Event Route:

F->F^F->BFK^F->BFK|F

PROBABILITY:

0.0455

NODE183:

EVENT MATRIX:

Dispersal:0

Vicariance:0

Extinction:0

Event Route:

D->D^D->D|D

PROBABILITY:

1.0000

NODE184:

EVENT MATRIX:

Dispersal:0

Vicariance:0

Extinction:0

Event Route:

D->D^D->D|D

PROBABILITY:

1.0000

NODE185:

EVENT MATRIX:

Dispersal:0

Vicariance:0

Extinction:0

Event Route:

D->D^D->D|D

PROBABILITY:

1.0000

NODE186:

EVENT MATRIX:

Dispersal:0

Vicariance:0

Extinction:0

Event Route:

D->D^D->D|D

PROBABILITY:

1.0000

NODE187:

EVENT MATRIX:

Dispersal:0

Vicariance:0

Extinction:0

Event Route:

D->D^D->D|D

PROBABILITY:

1.0000

NODE188:

EVENT MATRIX:

Dispersal:0

Vicariance:0

Extinction:0

Event Route:

D->D^D->D|D

PROBABILITY:

1.0000

NODE189:

EVENT MATRIX:

Dispersal:0

Vicariance:0

Extinction:0

Event Route:

D->D^D->D|D

PROBABILITY:

1.0000

NODE190:

EVENT MATRIX:

Dispersal:0

Vicariance:0

Extinction:0

Event Route:

D->D^D->D|D

PROBABILITY:

1.0000

NODE191:

EVENT MATRIX:

Dispersal:0

Vicariance:0

Extinction:0

Event Route:

D->D^D->D|D

PROBABILITY:

1.0000

NODE192:

EVENT MATRIX:

Dispersal:0

Vicariance:0

Extinction:0

Event Route:

D->D^D->D|D

PROBABILITY:

1.0000

NODE193:

EVENT MATRIX:

Dispersal:0

Vicariance:0

Extinction:0

Event Route:

D->D^D->D|D

PROBABILITY:

1.0000

NODE194:

EVENT MATRIX:

Dispersal:0

Vicariance:1

Extinction:0

Event Route:

DE->E|D

PROBABILITY:

1.0000

NODE195:

EVENT MATRIX:

Dispersal:0

Vicariance:0

Extinction:0

Event Route:

D->D^D->D|D

PROBABILITY:

1.0000

NODE196:

EVENT MATRIX:

Dispersal:0

Vicariance:0

Extinction:0

Event Route:

D->D^D->D|D

PROBABILITY:

1.0000

NODE197:

EVENT MATRIX:

Dispersal:1

Vicariance:0

Extinction:0

Event Route:

D->D^D->DE^D->D|DE

PROBABILITY:

1.0000

NODE198:

EVENT MATRIX:

Dispersal:0

Vicariance:1

Extinction:0

Event Route:

DF->D|F

PROBABILITY:

1.0000

NODE199:

EVENT MATRIX:

Dispersal:3

Vicariance:0

Extinction:0

Event Route:

F->F^F->DFHI^F->FHI|DF

PROBABILITY:

1.0000

NODE200:

EVENT MATRIX:

Dispersal:1

Vicariance:0

Extinction:0

Event Route:

F->F^F->FI^F->FI|F

PROBABILITY:

1.0000

NODE201:

EVENT MATRIX:

Dispersal:4

Vicariance:0

Extinction:0

Event Route:

H->H^F^G^H->FGH^F^G^H->FGH|FGH

PROBABILITY:

0.5000

NODE202:

EVENT MATRIX:

Dispersal:0

Vicariance:0

Extinction:0

Event Route:

H->H^H->H|H

PROBABILITY:

0.2500

NODE203:

EVENT MATRIX:

Dispersal:0

Vicariance:1

Extinction:0

Event Route:

FH->H|F

PROBABILITY:

0.2500

NODE204:

EVENT MATRIX:

Dispersal:0

Vicariance:0

Extinction:0

Event Route:

H->H^H->H|H

PROBABILITY:

1.0000

NODE205:

EVENT MATRIX:

Dispersal:0

Vicariance:0

Extinction:0

Event Route:

H->H^H->H|H

PROBABILITY:

1.0000

NODE206:

EVENT MATRIX:

Dispersal:1

Vicariance:0

Extinction:0

Event Route:

H->H^H->HJ^H->H|HJ

PROBABILITY:

1.0000

NODE207:

EVENT MATRIX:

Dispersal:0

Vicariance:0

Extinction:0

Event Route:

H->H^H->H|H

PROBABILITY:

1.0000

NODE208:

EVENT MATRIX:

Dispersal:2

Vicariance:0

Extinction:0

Event Route:

H->H^H^J->HJ^H^J->HJ|HJ

PROBABILITY:

1.0000

NODE209:

EVENT MATRIX:

Dispersal:0

Vicariance:0

Extinction:0

Event Route:

H->H^H->H|H

PROBABILITY:

1.0000

NODE210:

EVENT MATRIX:

Dispersal:0

Vicariance:0

Extinction:0

Event Route:

H->H^H->H|H

PROBABILITY:

1.0000

NODE211:

EVENT MATRIX:

Dispersal:0

Vicariance:1

Extinction:0

Event Route:

HJ->J|H

PROBABILITY:

1.0000

NODE212:

EVENT MATRIX:

Dispersal:2

Vicariance:0

Extinction:0

Event Route:

H->H^H->FHJ^H->HJ|FH

PROBABILITY:

0.1250

NODE213:

EVENT MATRIX:

Dispersal:0

Vicariance:0

Extinction:0

Event Route:

F->F^F->F|F

PROBABILITY:

1.0000

NODE214:

EVENT MATRIX:

Dispersal:2

Vicariance:0

Extinction:0

Event Route:

F->F^F->ABF^F->ABF|F

PROBABILITY:

1.0000

NODE215:

EVENT MATRIX:

Dispersal:0

Vicariance:1

Extinction:0

Event Route:

FH->F|H

PROBABILITY:

0.1250

NODE216:

EVENT MATRIX:

Dispersal:3

Vicariance:0

Extinction:0

Event Route:

F->F^F->ABFH^F->ABF|FH

PROBABILITY:

0.5000

NODE217:

EVENT MATRIX:

Dispersal:0

Vicariance:1

Extinction:0

Event Route:

FH->H|F

PROBABILITY:

1.0000

NODE218:

EVENT MATRIX:

Dispersal:1

Vicariance:0

Extinction:0

Event Route:

F->F^F->FH^F->FH|F

PROBABILITY:

1.0000

NODE219:

EVENT MATRIX:

Dispersal:3

Vicariance:0

Extinction:0

Event Route:

AFHI->AFHI^F->ABCFHI^F->ABCFIH|F

PROBABILITY:

0.0588

NODE220:

EVENT MATRIX:

Dispersal:4

Vicariance:0

Extinction:0

Event Route:

ABC->ABC^B->ABCFGH^B->B|ABCFGH

PROBABILITY:

0.0588

NODE221:

EVENT MATRIX:

Dispersal:3

Vicariance:0

Extinction:0

Event Route:

ABCH->ABCH^A->ABCFHI^A->ABC|AFHI

PROBABILITY:

0.0002

===================

Dispersal Between Areas:

A->B:2.75

A->C:0.25

A->F:0.5833334

A->G:0.3333333

A->H:0.3333333

A->I:0.25

B->A:1.083333

B->C:0.3333333

B->F:0.5833334

B->G:0.3333333

B->H:0.3333333

B->I:0.8333333

C->A:0.25

C->B:2.5

C->D:0.3333333

C->E:0.3333333

C->F:0.5833334

C->G:0.3333333

C->H:0.8333333

C->I:0.5

D->E:1.333333

F->A:16.08333

F->B:19.75

F->C:11.58333

F->D:5.333333

F->E:0.3333333

F->G:4

F->H:18.5

F->I:14.58333

F->J:1

F->K:2

H->A:0.25

H->B:0.25

H->C:0.25

H->D:4

H->F:3.25

H->G:8

H->I:4.5

H->J:9

I->B:0.25

I->C:0.25

I->D:0.3333333

K->A:0.3333333

K->B:0.5

K->C:0.3333333

K->I:0.3333333

K->L:6

Speciation Within Areas:

A:7

B:8

C:8

D:16

F:51

G:4

H:21

I:5

J:2

K:9

L:1

Dispersal Table:

from to within

A 4.50 18.00 7

B 3.50 26.00 8

C 5.67 13.00 8

D 1.33 10.00 16

E 0.00 2.00 0

F 93.17 5.00 51

G 0.00 13.00 4

H 29.50 20.00 21

I 0.83 21.00 5

J 0.00 10.00 2

K 7.50 2.00 9

L 0.00 6.00 1

===================

Global Cost:

Global Dispersal: 153

Global Vicariance: 13

Global Extinction: 2
